# Tuning Anti-Biofilm Activity of Manganese(II) Complexes: Linking Biological Effectiveness of Heteroaromatic Complexes of Alcohol, Aldehyde, Ketone, and Carboxylic Acid with Structural Effects and Redox Activity

**DOI:** 10.3390/ijms22094847

**Published:** 2021-05-03

**Authors:** Agnieszka Jabłońska-Wawrzycka, Patrycja Rogala, Grzegorz Czerwonka, Sławomir Michałkiewicz, Maciej Hodorowicz, Katarzyna Gałczyńska, Beata Cieślak, Paweł Kowalczyk

**Affiliations:** 1 Institute of Chemistry, Jan Kochanowski University of Kielce, 7 Uniwersytecka Str., 25-406 Kielce, Poland; patrycja.rogala@ujk.edu.pl (P.R.); slawomir.michalkiewicz@ujk.edu.pl (S.M.); 2 Institute of Biology, Jan Kochanowski University of Kielce, 7 Uniwersytecka Str., 25-406 Kielce, Poland; gczerwonka@ujk.edu.pl (G.C.); katarzyna.galczynska@ujk.edu.pl (K.G.); 3 Faculty of Chemistry, Jagiellonian University, 2 Gronostajowa Str., 30-387 Kraków, Poland; hodorowm@chemia.uj.edu.pl; 4 Labsoft Sp. z o.o., 469 Puławska Str., 02-844 Warszawa, Poland; beatacieslak@o2.pl; 5 Department of Animal Nutrition, The Kielanowski Institute of Animal Physiology and Nutrition, Polish Academy of Sciences, 3 Instytucka Str., 05-110 Jabłonna, Poland; p.kowalczyk@ifzz.pl

**Keywords:** manganese(II) complexes, crystal structure, HS surface analysis, antibacterial and anti-biofilm activity, structure–activity relationship, inhibition effect of catalase

## Abstract

The constantly growing resistance of bacteria to antibiotics and other antibacterial substances has led us to an era in which alternative antimicrobial therapies are urgently required. One promising approach is to target bacterial pathogens using metal complexes. Therefore, we investigated the possibility of utilizing series of manganese(II) complexes with heteroaromatic ligands: Alcohol, aldehyde, ketone, and carboxylic acid as inhibitors for biofilm formation of *Pseudomonas aeruginosa*. To complete the series mentioned above, Mn-dipyCO-NO_3_ with dipyridin-2-ylmethanone (dipyCO) was isolated, and then structurally (single-crystal X-ray analysis) and physicochemically characterized (FT-IR, TG, CV, magnetic susceptibility). The antibacterial activity of the compounds against representative Gram-negative and Gram-positive bacteria was also evaluated. It is worth highlighting that the results of the cytotoxicity assays performed (MTT, DHI HoloMonitorM4) indicate high cell viability of the human fibroblast (VH10) in the presence of the Mn(II) complexes. Additionally, the inhibition effect of catalase activity by the complexes was studied. This paper focused on such aspects as studying different types of intermolecular interactions in the crystals of the Mn(II) complexes as well as their possible effect on anti-biofilm activity, the structure–activity relationship of the Mn(II) complexes, and regularity between the electrochemical properties of the Mn(II) complexes and anti-biofilm activity.

## 1. Introduction

The problem of global antibiotic resistance among bacteria is growing to an unimaginable extent in the 21st century. Microbial resistance is a consequence of not only the massive use of antibiotics, including their often unjustified or excessive use, but also of the ability of pathogens to adapt to different conditions and develop self-defense mechanisms such as living in biofilms [1,2,3,4]. Biofilms include bacterial microcolonies adhering to the surface and surrounded by a viscous extracellular matrix. When attached, bacteria reproduce and anchor in fairly complex structures that appear to enable communication and transfer of nutrients, waste, and signaling compounds (*quorum sensing*) [3]. Bacterial biofilms are extremely difficult to eliminate with conventional antibiotics and therefore pose a huge burden to healthcare. *Pseudomonas aeruginosa* is a particularly dangerous microorganism—an opportunistic pathogen that shows great ease in biofilm formation. This bacterium has become extremely dangerous in the hospital environment over the past few decades [5]. *P. aeruginosa* is responsible for causing various infections, often of a complicated course, in people with weakened immunity, e.g., cystic fibrosis, oncological, or transplant patients. Hence, there is an urgent need to look for new strategies to develop new compounds to combat multi-drug-resistant and infection biofilms. Researchers are using various approaches to identify new antibiotics or new compounds with antimicrobial activity. Various inhibitors of different pathways, antimicrobial peptides [6,7,8], as well as organic and inorganic synthetic compounds [9,10,11,12,13] with antibacterial activity, have been identified and studied. After the success of transition metal coordination compounds as antibacterial and anticancer agents (Ag, Cu, Ru, Au, Zn, Mn) [14,15,16,17,18], the use of this group of compounds brings enormous potential in medicine, biotechnology, and pharmacy applications. 

Mn, along with Cu, Fe, and Zn, is one of the most investigated metal ions among other transition metals and holds importance due to its significant role in the various biological activities [19,20]. It is included in enzymes (SOD, CAT) that play an important role as a defense system against reactive oxygen species. Interestingly, some experts believe (Department and Institute of Hygiene AM Wrocław) that a manganese compound in the right concentration has an antioxidant effect. When it occurs in excess, its effect is the opposite. It multiplies free radicals and inhibits the activity of antioxidant enzymes. Several reports of the medicinally tested Mn complexes showing antibacterial and anticancer activity have been published earlier [20,21]. The above-mentioned aspects encouraged us to engage in the search for new anti-biofilm agents. Looking at the structure of the complexes studied so far, it seems that the presence of privileged structures promotes their significant biological activity. According to the reports, functional groups containing oxygen and sulfur donors have a potential role in fighting cancer cells and bacteria [22,23]. Additionally, imidazole and pyridine are well-known pharmacists whose derivatives are currently used against various diseases [24,25,26,27,28]. In our research, we employed the N,O-donor ligands, in which imidazole and pyridine were substituted by -OH, -CHO, and -COOH functional groups. A series of manganese(II) complexes with the N-heteroaromatic alcohol, aldehyde, and carboxylic ligands were synthesized by our group previously [29,30,31,32,33]. These ligands allow coordination to a metal ion by nitrogen and oxygen atoms. The complement of this group of compounds is the Mn(II) complex with dipyridin-2-ylmethanone (Mn-dipyCO-NO_3_). This manuscript presented its structure and characteristics as well as the preparation of the complex.

Herein, we presented the biological efficacy of a series of manganese(II) complexes in the inhibition of bacterial biofilm formation. In this regard, our studies focused on the following aspects: (i) Evaluation of antibacterial and anti-biofilm activities; (ii) cytotoxicity assays for a series of Mn(II) complexes using the MTT test and digital holographic imaging (DHI) platform HoloMonitorM4 to evaluate the effects of the complexes examined on cell viability in the primary human fibroblast (VH10) as a model; (iii) inhibition effect on CAT activity by a series of Mn(II) complexes; (iv) structure–activity relationship of a series of Mn(II) complexes supported by heteroaromatic alcohol, aldehyde, ketone, and acid; (v) regularity between electrochemical properties of Mn(II) complexes and anti-biofilm activity.

## 2. Results and Discussion

### 2.1. Synthesis and Characterization of Mn-dipyCO-NO_3_

In our previous studies, we reported the syntheses and characterization of the manganese(II) complexes with N-heteroaromatic alcohol, aldehyde, and carboxylic acid ligands (Mn-pyOH-NO_3_, [Mn-pyOH-SO_4_]_n_, Mn-imCHO-NO_3_, Mn-imCHO-Cl, Mn-pyCOOH-H_2_O, [Mn-pyCOOH-H_2_O]_n_; where pyOH–2-hydroxymethylpyridyne, imCHO–5(4)-carbaldehyde-4(5)-methylimidazole, pyCOOH–pyridine-2,3-dicarboxylic anion) [29,30,31,32,33]. In this paper, we focused on obtaining the Mn(II) complex with a ketone to complete the series of Mn(II) compounds. The reaction of Mn(NO_3_)_2_*·*4H_2_O and dipyridin-2-ylmethanone (dipyCO) in CH_3_CN led to the formation of a molecular complex formulated as Mn-dipyCO-NO_3_. An interesting behavior of the ligand depending on the type of solvent used during the synthesis was observed. The addition of the metal salt and ligand solutions obtained by dissolving in the mixture H_2_O:CH_3_CN or H_2_O:EtOH resulted in a color change to olive green, and as a result, obtaining an olive precipitate of the *gem*-diol complex with Mn(II) ion (IR spectra were made confirming the presence of OH groups). However, the use of acetonitrile as a solvent for the synthesis caused the solution obtained after mixing the metal and ligand solutions to turn yellow. After a week, orange crystals of the dipyCO complex with Mn(II) ions were isolated. Our goal was to examine the latter of the described products. Therefore, no analyses were undertaken for the *gem*-diol complex except for the recorded IR spectra.

The FT-IR spectrum of the Mn-dipyCO-NO_3_ indicates a ketone form of the ligand in the complex. The characteristic FT-IR bands of the corresponding organic molecule (dipyCO) were observed in the IR spectra of the complex. In the region of the wavenumbers higher than 2900 cm^−1^, the weak intensive maxima of the *ν*_C_*_−_*_H_ aromatic stretching vibrations were detected at 3057 and 3124 cm^−1^. The intensive *ν*_C = N_ and *ν*_C = C_ aromatic vibrations in the spectrum of the complex were detected at 1587, 1568, 1468, 1439, and 1429 cm^−1^ (Table S1). The vibration was shifted by ca 10–20 cm^−1^ with respect to the spectrum of the free organic molecule, thus suggesting changes in the vicinity of heterocyclic nitrogen connected with coordination to manganese. The band assigned to the carbonyl group, present in the spectrum of the free ligand (1680 cm^−1^), was also shifted, but towards higher energies (Table S1), and it was split. This behavior confirmed the N,O-coordination mode of the ligand. The nitrate frequencies cited in Table S1 arose from the spectrum obtained by the ATR technique, which deserves comments. Interestingly, it is well known that pressing a sample affects the nitrate coordination observed in the FT-IR spectrum [34,35]. The interpretation is particularly difficult when nitrates appear as counterions in the structure of the complex, or the asymmetry of the bidentate nitrates is very high (anisobidentate). In the ATR spectra of the Mn(II) complex, the bands at 1448 and 1298 cm^−1^ were assigned to the ν_1_(A1) and ν_4_(B2) stretching modes of the nitrate ligands, respectively [36]. Their separation was large (~150 cm^−1^) in accordance with the bidentate character of the nitrate groups [36]. The existence of the bidentate nitrate was also deduced from the appearance of the strong ν_2_, ν_5_, and ν_6_ stretching modes. It should be mentioned at this point that the highest frequency NO stretching mode belongs to the A1 species in the complexes containing bidentate nitrate ligands. The value ∆ for the ν_1_ + ν_4_ splitting in the FT-IR spectrum of the complex listed in Table S1 was over 25. According to Lever’s criteria [37], the value indicates bidentate nitrate coordination. Regarding the far-IR region, new bands, as compared to the spectrum of the uncoordinated organic ligand, observed at 228 and 219 cm^−1^, can be attributed to the ν_Mn−N_ vibrations. The prominent ν_Mn−O_ vibrations were also observed at 368 and 331 cm^−1^ in the spectrum of the Mn(II) complex. Thus, this supports the conclusions about the coordination of the organic molecules to the central ion.

To study the composition of the complex and the Mn-dipyCO-NO_3_ stability, thermal behavior was analyzed by the TG and DTG techniques. Figure S1 shows the TG and DTG curves of the complex. From the TG and DTG curves, it can be observed that a three-step thermal degradation process exists. For this complex, the first stage of degradation began at 448 K and terminated at 540 K, and the observed mass loss was 44.58%, which corresponds to the loss of the two C_6_H_4_N molecules. The second stage of degradation, between 540 and 628 K, resulted in 16.58% weight loss, and it is attributed to the release of the two nitrates. The third stage of degradation occurred in the temperature range of 628–1073 K. In the last stage, the next ligand fragments were lost from the complex. All the mass losses were accompanied by exothermic effects. The stages of degradation were summarized in Table 1. It can be clearly seen (Table 1) that the mass losses obtained from the TG curves and those calculated for the corresponding molecules and final decomposition are in good agreement for all the decomposition steps. The thermal degradation studies of the Mn(II) complex indicated that this complex was thermally stable up to 448 K, and continued degrading up to 1073 K. The decomposition ended with 83.93% mass loss at 1073 K, with a blackish-colored solid. Pyrolusite (MnO_2_), as the final product, was confirmed from the XRD patterns and identified on the basis of ICDD using the XRAYAN package [38].

**Figure 1:**
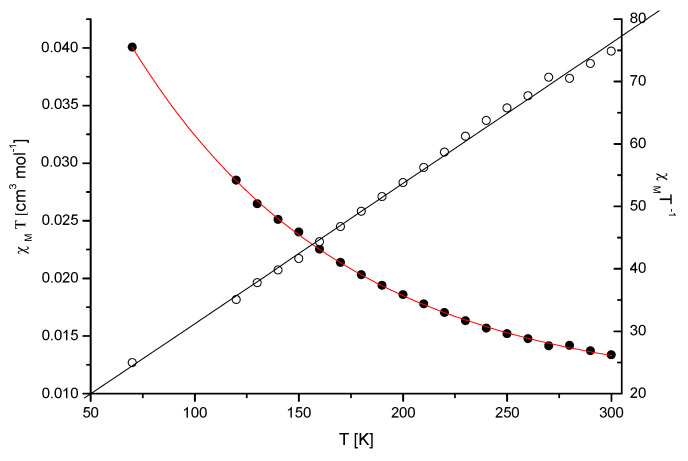
Plot of χ_M_ T vs. T for the crystalline sample of Mn-dipyCO-NO_3_. The solid lines were theoretical ones.

The magnetic susceptibility of the Mn(II) complex (Mn-dipyCO-NO_3_) was measured over the range of 70–300 K (Figure 1). The measured values obey the Curie–Weiss law, suggesting paramagnetic properties. The magnetic moment values, experimentally determined at 70–300 K for the compound, changed from 4.94 to 5.69 MB. The value of the room temperature magnetic moment of Mn-dipyCO-NO_3_ is equal to 5.67 MB, and it is indicative of high-spin Mn(II) ion. This value is very close to the spin- only value for the Mn^2+^ ion calculated from the equation µ_eff_ = [4S(S + 1)]^1/2^ in the absence of the magnetic field. The high-spin Mn(II) ion possesses a half-filled 3D shell and thus the coordination geometry of its complex is not subject to ligand field effects and are determined mainly by the chelating power and geometrical arrangement of the ligand.

### 2.2. Description of the Molecular and Crystal Structure

**Figure 2:**
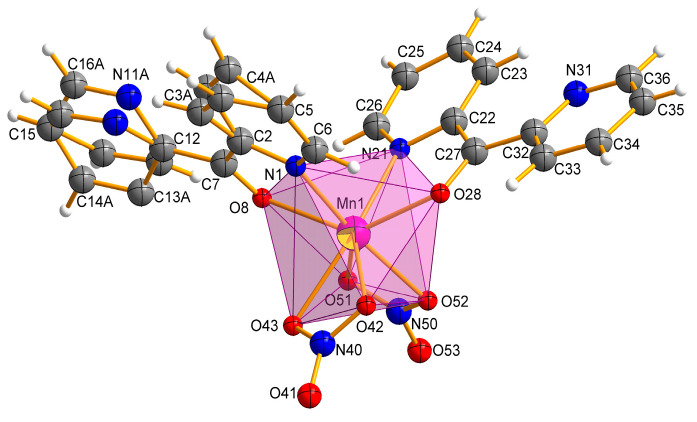
Molecular structure with the atom numbering scheme and coordination polyhedron of Mn-dipyCO-NO_3_.

The Mn(II) complex crystallizes in a triclinic space group P$\bar{1}$. The molecular structure of the complex Mn-dipyCO-NO_3_ is shown in Figure 2. The selected bond lengths and angles are listed in Table S2. The manganese environment is eight-coordinated with the two nitrate ions and two dipyCO molecules. The complexes with CN = 8 are very rare. Both bidentate nitrate and organic ligands are arranged in *cis* position. The manganese environment adopts distorted *pseudo*-dodecahedral geometry resulting from the positions of the organic and inorganic ligands, as they are not symmetrical or equivalent. In general, the deviation from the ideal dodecahedral geometry is shown by the bond distances resulting from the size of the chelating rings.

The literature data indicate that the coordination possibility of the dipyCO is rich, and the ligand can form connections with metal ions while remaining in the ketone or *gem*-diol form (obtained by the nucleophilic addition of water or alcohol at the carbonyl carbon atom) with the formation of mononuclear, dinuclear, and polynuclear compounds. In the ketone form, the dipyCO can coordinate as a bidentate ligand to the metal ion via one of the two modes: Either through the two pyridyl N atoms or through one pyridyl N atom and the carbonyl O atom. In the case of the analogous dipyCO complexes, in the majority of cases, the ligand was in the N,N-coordination mode. This coordination type was observed upon the coordination of the dipyCO to the metal ions such as Zn(II) [39], Pt(IV) [40,41], Ru(II) [42]. The complexes with the N,O-coordination were in the minority. The coordination took place when the metal ions such as Ag(I) [43], Y(III) [44], and Rh(III) [45] were present in the complexes. Interestingly, Toyama et al. [46] have reported that the *trans*(Cl),*cis*(S)-[RuCl_2_(dpk-κ^2^N,O)(dmso-S)_2_] complex obtained by using water solution (r.t.) exhibits the *N*,O-coordination mode, whereas synthesis in EtOH-DMSO mixture (at reflux condition) afforded cis(Cl),cis(S)-[RuCl_2_-(dpk-κ^2^N,N’)(dmso-S)_2_], in which a ketone was coordinated in the N,N-chelating manner. The authors stated that the N,O-coordination mode of the dipyCO was kinetically favorable due to a small O donor atom of the carbonyl group, but the N,N-coordination mode was more thermodynamically stable. 

We suggests that the type of coordination can be influenced by the ionic radius of the metal. Thus, for the first group of the literature complexes with the N,N-coordination mode of the dipyCO, the ionic radii were in the range of 74–76.5 pm, while for the second group of the complexes in the N,O-coordination fashion, the ionic radii were in the range of 80.5–116 pm. As the value of the ruthenium ion radius (88 pm) was in the middle of the ranges indicated, the ligand could coordinate to the ruthenium ion via the first or the second mode, in proper synthetic condition. In our paper, organic ligands act as chelating, coordinating through a pyridyl N atom and a carbonyl O atom. The presence of the Mn(II) ion with the ionic radius of 110 pm seems to confirm our suggestion. All the Mn-ligand distances, such as Mn–N 2.2711(1), 2.3176(2) Å, Mn–O 2.3032(1), and 2.3137(1) Å, are normal and comparable with the distances in other Mn(II) complexes containing the N,O-heteroaromatic ligands [29,31,47,48]. 

In the manganese complex, one of the organic ligands is disordered over two occupation sites: A and B, with refined site-occupation factors of 0.34:0.66. However, the other ligand molecule is fully ordered. The planar pyridine rings of the dipyCO are twisted differently around the planar ketone subunit by twisted angles 30°(8°) and −161°(−3°). The plane of the rings 1 and 2 are twisted relative to each other by 17° with their N centers approaching anti-configuration, which avoids any repulsive interaction between the nitrogen N1 and N11 (N21 and N31). The two oxygen atoms of the bidentate nitrate ion O(42) (O(51)) and O(43) (O(52)) bind to the Mn(II) asymmetrically with Mn-O distances in the range of 2.2814(1)–2.4245(1) Å. Both nitrate ions can be classified as bidentate according to the structural criteria used for assigning coordination modes (Table 2).

**Figure 3:**
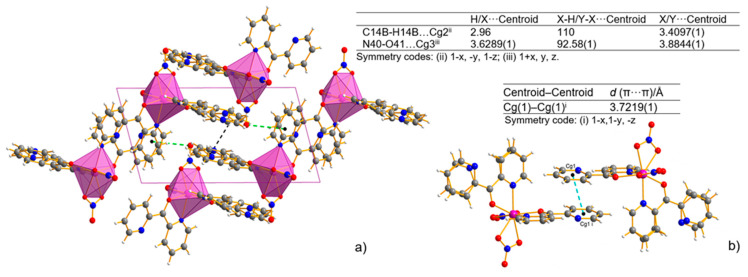
Packing view of the coordination polyhedral system forming a rhombic 3D network and the geometry of C–H⋯π (black line) and N–O⋯π (green line) interactions (X-H⋯π and Y-X⋯π interactions, view along [010] direction)—(**a**), and π⋯π stacking interaction—(**b**) for Mn-dipyCO-NO_3_. Cg(1) denotes the gravity ring center of N(31)-C(32)-C(33)-C(34)-C(35)-C(36); Cg(2) denotes the gravity ring center of C(12)-N(11B)-C(16B)-C(15)-C(14B)-C(13B); Cg(3) denotes the gravity ring center of N(21)-C(22)-C(23)-C(24)-C(25)-C(26).

The analysis of the crystal packing revealed only a weak intermolecular interaction without any classical H-bonds (Table S3). The shortest intermolecular distance is determined for C–H⋯N (D⋯A distance varies from 2.885(2) to 2.911(6) Å). The crystal structure is also stabilized by C–H⋯O weak intermolecular hydrogen bonds. The D⋯A distance varies from 3.101(2) to 3.489(2) Å, and the D–H⋯A angle varies from 116.9 to 147.7°. The intermolecular hydrogen bonds linking the N atom of the uncoordinated pyridyl ring or the O atom of the nitrate ions and the C atom of the adjacent pyridyl ring are observed. The intermolecular π–π stacking interaction further stabilizes the mononuclear structure in the complex. The two uncoordinated pyridyl rings (Figure 3a) are approximately parallel to each other. The distance between the centroids of the pyridyl rings is 3.7219(1) Å, indicating normal intermolecular π–π interaction. Non-covalent interactions that involve the π system were also found out in the C-H⋯π and N-O⋯π interactions (Figure 3b).

### 2.3. Hirshfeld Surface Analysis of the Series Mn(II) Complexes

**Figure 4:**
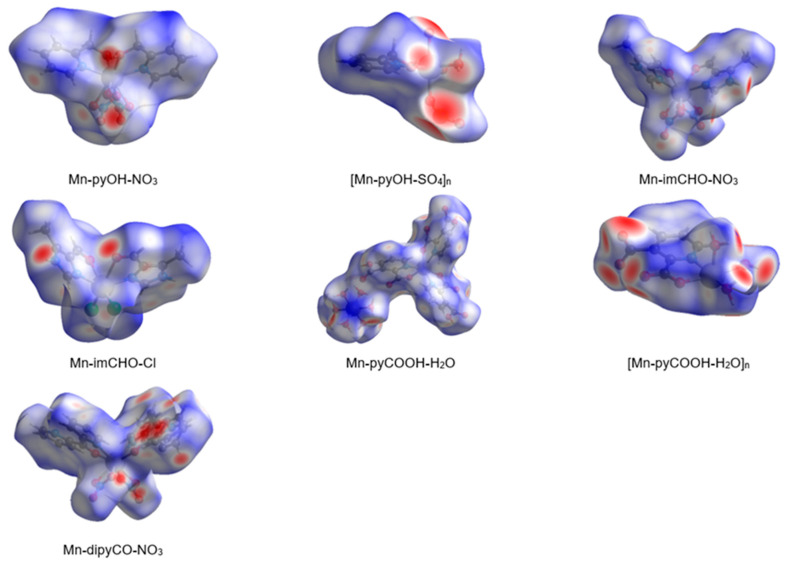
The Hirshfeld surfaces of series of the Mn(II) complexes mapped with 3D *d*_norm_ (with transparency enabled).

In the last decade, the analysis of molecular crystal structures using Hirshfeld surfaces has rapidly gained in popularity. HS analysis provides a convenient means of studying different types of intermolecular interactions in crystals, because this tool enables these interactions to be interpreted by visualization [49]. This applies especially to the identification of close contacts deemed to be important—and to view molecules as “a whole”. The molecular Hirshfeld surface of the Mn(II) complexes were presented in Figure 4. Three-dimensional (3D) Hirshfeld surface maps were obtained using red–white–blue *d*_norm_ surface maps (surface resolution −0.5 to 1.6 Å), where red indicates shorter contacts with negative *d*_norm_ values, white indicates close van der Waals contacts with zero *d*_norm_ values, and blue indicates longer contacts with positive *d*_norm_ values. A shape index provided a measure of the “shapes” of molecules in lattices, enabled complementarity between molecules to be identified, and provided π–π interaction information. Some significant π–π interactions were observed for all of complexes, (red and blue triangles represented π–π stacking). 

**Figure 5:**
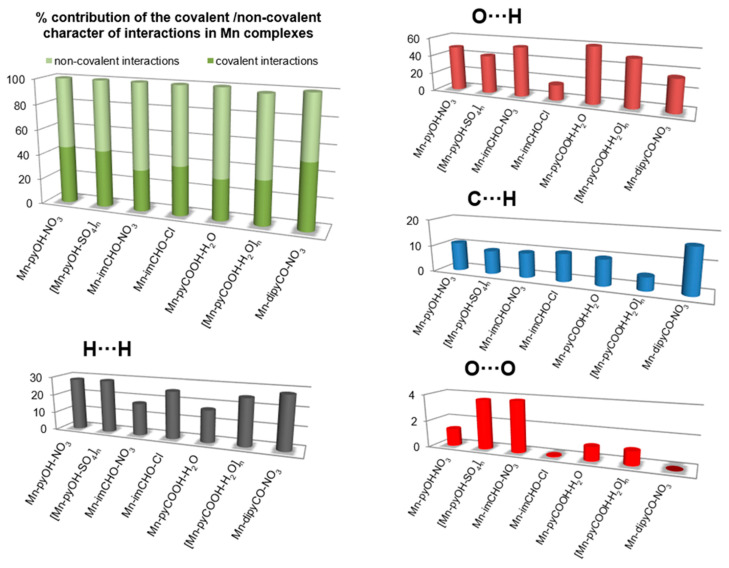
The plots for the most significant intermolecular interaction within the series of the Mn(II) complexes showing percentages of contacts contributing to the total HS area.

The 2D fingerprint plots obtained by Hirshfeld surface analysis were also studied (Figure S2). Main reciprocal intermolecular interactions (O⋯H, H⋯H, C⋯H) were obtained using a 2D fingerprint plot and 3D *d*_norm_ surfaces of the complexes (Figure 5). The five spikes were observed in the 2D fingerprint plots corresponding to O⋯H, C⋯H, and H⋯H reciprocal close contacts. Figure 5 shows O⋯H interactions contributed most (16.5–53.8%) to the total Hirshfeld surfaces, as indicated by the two spikes in the 2D fingerprint plot and the red circles in the 3D *d*_norm_ promolecular map. Reciprocal H⋯H interactions contributed second most (17.2–28.6%) to the total Hirshfeld surface, and were indicated in the 2D finger print plot as the middle spike and in the 3D *d*_norm_ surface as the blue colored region (for Mn-pyOH-NO_3_, [Mn-pyOH-SO_4_,]_n_, Mn-imCHO-NO_3_, Mn-imCHO-Cl, [Mn-pyCOOH-H_2_O]_n_), or as red circle (for Mn-dipyCO-NO_3_, Mn-pyCOOH-H_2_O). Reciprocal C⋯H intermolecular contacts appeared as two spikes on the top left and bottom right in the 2D fingerprint plots and as a blue colored region in the 3D *d*_norm_ promolecular surface, which contributed from 4.9 to 16.4% to the total Hirshfeld surface, for the Mn complexes. 

The interesting feature of analysis is associated with the O⋯O interactions. Although these contacts are interpreted as a minor (range of 0.1–3.8%), it is deemed as important for biological activity. The percentage of contribution of the O⋯O interaction is increased in the following order for the Mn complexes: Mn-dipyCO-NO_3_ < Mn-imCHO-Cl < Mn-pyCOOH-H_2_O < [Mn-pyCOOH-H_2_O]_n_ < Mn-pyOH-NO_3_ < [Mn-pyOH-SO_4_]_n_ < Mn-imCHO-NO_3_. The row is surprisingly consistent with that of the anti-biofilm activity discussed in biological studies section.

### 2.4. Electrochemical Studies (CV and DPV Methods)

The solutions containing 1.0 mM of the Mn-pyOH-NO_3_, Mn-imCHO-NO_3_, Mn-imCHO-Cl, and Mn-dipyCO-NO_3_ in acetonitrile and CH_3_CN/glacial CH_3_COOH were used for electrochemical studies. In all cases, the CV curves were recorded from the initial potential −1.0 V vs. Ag/AgCl (3M KCl). The use of acetonitrile as a solvent resulted in a too-narrow measuring range for the tested manganese(II) complexes. Therefore, the oxidation curves (Figure S3) that were observed for the Mn-pyOH-NO_3_ did not take the shape of a peak. The poor peak shape made a precise quantitative analysis impossible. Based on the recorded curves, it can be seen that oxidation proceeded in an irreversible manner, as evidenced by the shifting of the anodic signal towards higher potentials as the scan rate increases. A very weak signal from the reduction of the oxidation of the final product could be seen at approximately 0.6 (12.5) and 0.2 V (50 mVs^−1^, Figure S3). The recorded cathodic signal, as in the case of the anodic signal, was shifted with the increase of the scan rate, but a shift towards lower potential values was observed (which was a characteristic feature of irreversible processes). The intensity of the currents for these signals also increased with the increase of the scan rate, which proved their connection with the oxidation process. Attempts were made to estimate the relevant electrochemical data for the scanning rate of 4.2 mVs^−1^. The obtained values of *E*_pa_ and *E*_pa/2_ were 1.55 and 1.29 V, respectively. The difference between the above-mentioned values (0.260 V) significantly exceeded the value predicted for a reversible process (0.0565/n V), which confirmed the irreversible nature of the electron exchange process (Mn(II)→Mn(III)). In the case of irreversible processes, the criterium *E*_pa_ − *E*_pa/2_ = 0.0477/nα V was satisfied [50]. For α = 0.3, the electron exchange number was 0.6, so it could be assumed with a high probability that one electron was transferred.

**Figure 6:**
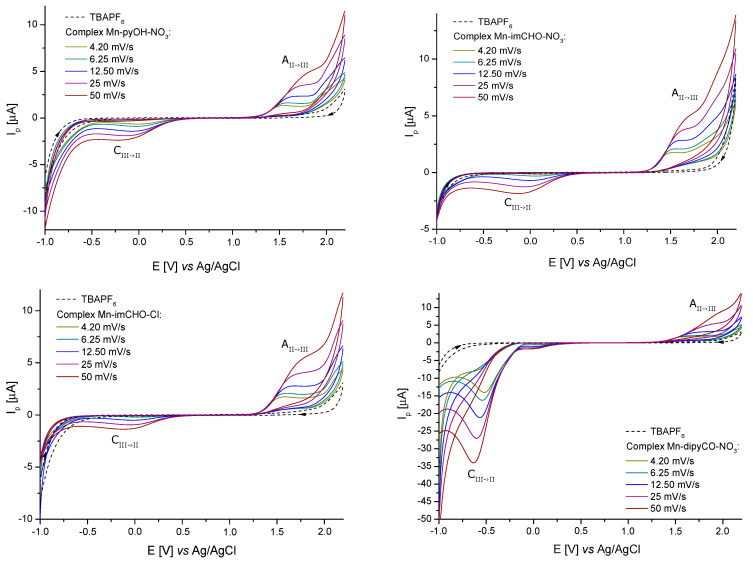
Cyclic voltammograms of the Mn-pyOH-NO_3_, Mn-imCHO-NO_3_, Mn-imCHO-Cl, and Mn-dipyCO-NO_3_ recorded at scan rates from 4.20 to 50 mV s^−1^ in CH_3_CN/glacial CH_3_COOH solution containing 0.1 M TBAPF_6_ (CV conditions: BDDE, Ø = 3 mm, T = 25 °C).

For better results, the measuring range of an electrochemical window was extended to 2.2 V by using an acetonitrile/glacial acetic acid mixture as a solvent. Figure 6 shows the cyclic voltammograms obtained from a 1.0 mM solution of the Mn-pyOH-NO_3_, Mn-imCHO-NO_3_, Mn-imCHO-Cl, and Mn-dipyCO-NO_3_. In all cases, one redox couple for a metal center was seen. For the selected complexes, one feature was observed in the anodic sweep, above 1.2 V (1.3 V for Mn-dipyCO-NO_3_). On the return sweep, a very weak signal was observed, in the range from 0.1 to −0.2 V. When the potential sweep was reversed at 1.0 V (just before the oxidation peak), the feature in the range from 0.1 to −0.2 V was absent. Clearly, the reduction and oxidation are linked (Table S4). It is possible that cathodic peak was associated with the reduction of the product of a chemical reaction following the initial oxidation or with the aftermaths of this reaction. 

A series of studies in which the potential scan rate varied from 4.2 to 50 mVs^−1^ were conducted using the solutions of the Mn-pyOH-NO_3_, Mn-imCHO-NO_3_, Mn-imCHO-Cl, and Mn-dipyCO-NO_3_ in the CH_3_CN/glacial CH_3_COOH mixture. However, the plot of the peak current vs. the square root of the scan rate showed a linear trend indicating that the oxidation of these complexes was diffusion controlled. The peak potential of the oxidation wave was observed to shift to a more positive value with an increasing scan rate. In contrast, the potential of the cathodic peak shifted to a more negative value with an increasing scan rate. Such behavior is typically associated with an electrochemically irreversible process. On this basis, the previous criterium is considered to indicate the exchange of one electron in all the recorded cases. In Table S4, the analysis of the electrochemical data is presented in detail. The electrochemical studies of the ligands (pyOH, imCHO) and TBAPF_6_ have shown that, in the selected potential range (−1.0 to 2.2 V), they were not electrochemically active (dashed lines in Figure 6). In contrast, the dipyCO in the Mn-dipyCO-NO_3_ was electrochemically active. For the Mn-dipyCO-NO_3_, an additional distinct signal at −0.63 V (for scan rate 50 mVs^−1^) was observed in cathodic direction. The cathodic peak was related to the reduction of the dipyCO ligand and it did not appear to have the reoxidation peak associated with it. The complementary CV and DPV studies for the dipyCO performed also confirmed these results (Figure S4). A visible change in the peak potential was observed for this signal with a changing scan rate (Figure 6). This process as such should be considered to be irreversible with respect to an electron transfer because the higher the scan rate, the more negative *E*_pc_ value. Collectively, the electrochemical data appeared to indicate that the initial reduction wave follows the mechanism: dipyCO $\overset{+e^{-}}{\to }$ $dipyCO^{•-}$.

Similar results were obtained by Paul et al. [51], who studied Rh(III) complexes. They postulated the irreversible nature of the dipyCO reduction. These results are substantially different from those observed by Bakir [52,53] for the Re(I) complex. They indicated that the reduction was highly reversible. Similarly, Tong et al. [54] observed the *quasi-*reversible reduction of the dipyCO ligand for the Cu(II) polynuclear complex.

For comparison, the cyclic voltammetry experiment was performed for manganese salt (Mn(NO_3_)_2_). Similarly to the complexes, the anodic signal appeared (Figure S5). However, it was a poorly shaped peak, the same as the cathodic peak. Therefore, the electrochemical data (Table S4) were estimated only. The reduction/oxidation couple appeared to be irreversible with respect to one electron exchange. 

**Figure 7:**
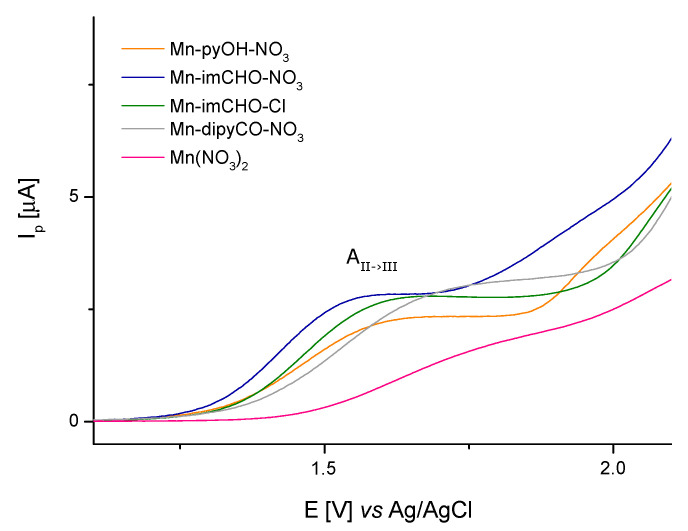
The voltametric curves (V = 6.25 mV s^−1^) showing the comparison of the anodic peaks related to oxidation of Mn(II)/Mn(III) for Mn-pyOH-NO_3_, Mn-imCHO-NO_3_, Mn-imCHO-Cl, and Mn-dipyCO-NO_3_.

Concluding, the anodic peak potentials related to the oxidation of the Mn(II)/Mn(III) increase in the following order for the manganese complexes: Mn-imCHO-NO_3_ < Mn-pyOH-NO_3_ < Mn-imCHO-Cl < Mn-dipyCO-NO_3_ (Figure 7; at scan rate 6.25 mVs^−1^). Thus, the presented row indicates a decrease in the reducing properties for the tested Mn(II) complexes. Thereby, the oxidation efficiency of the Mn(II) complexes results from the row mentioned above.

### 2.5. Antimicrobial Activity

#### 2.5.1. Antibacterial Activity

The manganese(II) complexes, manganese(II) salts, and ligands were screened for their bacteriostatic activity using the broth microdilution method by determining minimum inhibitory concentration. Antibacterial efficacy was compared with a commercial drug—streptomycin. The results of the MIC for the compounds tested are given in Table S5. Several Mn(II) complexes showed some antibacterial activity against planktonic cells of pathogens at the concentrations examined. The most effective compound tested against *S. aureus* in this study was the Mn-imCHO-NO_3_, with a MIC value of 399 μg/mL. In turn, the highest bacteriostatic activity against *P. aeruginosa* PAO1 was obtained for the complex [Mn-pyCOOH-H_2_O]_n_, in the concentration of 274 μg/mL. It was also found that the heteroaromatic ligands and Mn(II) salts were inactive against the bacterial strains used in our experiment.

#### 2.5.2. Antibiofilm Activity 

**Figure 8:**
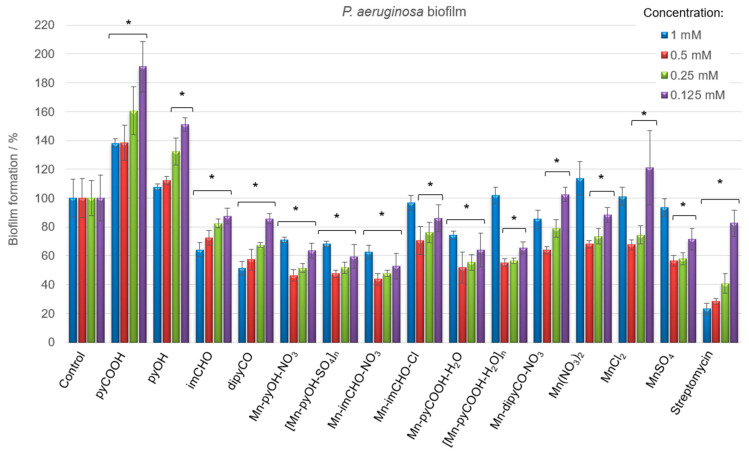
*P. aeruginosa* PAO1 biofilm formation in the presence of the Mn(II) complexes, free ligands, and Mn(II) salts (concentrations of compounds—1–0.125 mM). The absorbance of the control was considered to represent 100% of biofilm formation (results were considered significant when compared to control; * *p* < 0.05. Data are presented as mean ± SD, *n* = 4).

Having in view the differences in the physiology and susceptibility to antimicrobial substances of biofilm-embedded bacteria, the Mn(II) complexes were tested concerning their efficiency against the cells of *P. aeruginosa* biofilm (qualitatively and quantitatively). Biofilm biomass was assessed using a standard crystal violet assay, and data were expressed as the percentage. Figure 8 displays the results of the anti-biofilm activity of the Mn(II) complexes and the starting compounds used for their synthesis at various concentrations. It was observed that the free ligands and manganese(II) salts interact differently with the biofilm, with the effect being either inhibitory or stimulatory depending on the concentrations of the compounds. Among the ligands, the dipyCO exhibited the best effectiveness in inhibiting *P. aeruginosa* biofilm. At the concentration of 1 mM, the inhibition was 49%. 

As shown in Figure 8, very good results of the biofilm biomass reduction were obtained for the manganese(II) complexes. The most evident inhibitory effect was found for the compounds Mn-pyOH-NO_3_, [Mn-pyOH-SO_4_]_n_ and Mn-imCHO-NO_3_. The complexes were able to inhibit the biofilm formation, reducing the biomass by 53–56% at the concentration of 0.5 mM. A similar reduction outcome was obtained for the concentration of 0.25 mM (48–52%). The Mn-pyCOOH-H_2_O and [Mn-pyCOOH-H_2_O]_n_ also significantly reduced the biofilm growth of the *P. aeruginosa* PAO1 strain. The biofilm formation was inhibited by 48% (complex Mn-pyCOOH-H_2_O) and 45% (complex [Mn-pyCOOH-H_2_O]_n_) compared to an untreated control when the concentration of complexes was 0.5 mM. 

Overall, the Mn(II) complexes exhibit an improved activity in comparison with the free ligands and manganese(II) salts. The anti-biofilm study of the manganese(II) complexes investigated increases in the order of Mn-imCHO-Cl < Mn-dipyCO-NO_3_ < [Mn-pyCOOH-H_2_O]_n_ < Mn-pyCOOH-H_2_O < [Mn-pyOH-SO_4_]_n_ = Mn-pyOH-NO_3_ < Mn-imCHO-NO_3_, indicating that the compounds with NO_3_^-^ and SO_4_^2−^ ions are the most active from this series (the concentration of 0.5 and 0.25 mM). Interestingly, it should be noted that the highest and the lowest concentrations of the manganese compounds used (1 and 0.125 mM) have a much less anti-biofilm effect than the concentrations of 0.5 and 0.25 mM tested (Figure 8). In addition, the research group under the guidance of Nikodinovic–Runic and Senerovic noted that maximum anti-biofilm activity of the compound was reached at the middle-concentration tested [55]. Furthermore, it was observed that the activity of manganese complexes at the concentration of 0.125 mM is better than the activity of the antibiotic at the same concentration. 

**Figure 9:**
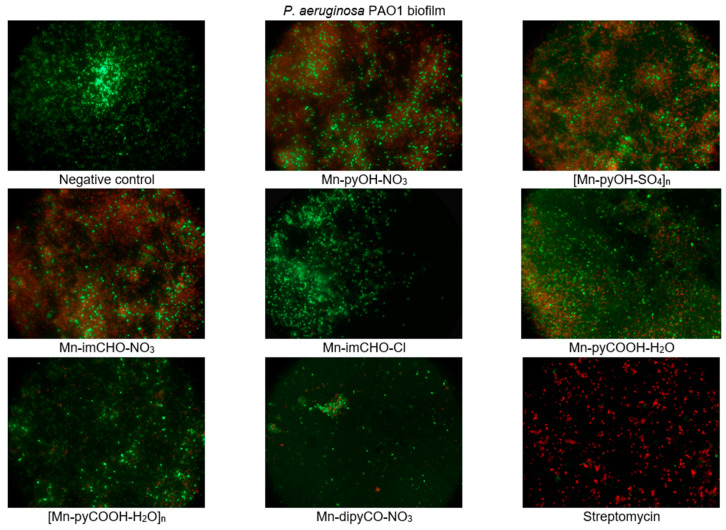
Epifluorescence microscopy images of *P. aeruginosa* PAO1 biofilm treated with 0.5 mM of the manganese complexes. Biofilm was stained with nucleic acid stains using the FilmTracer™ LIVE/DEAD Biofilm Viability kit (live cells are represented by the color green; dead cells are represented by the color red). The epifluorescence microscopy images were captured at 1000× magnification.

The effect of the manganese complexes (the concentration of 0.5 mM) on the cell viability of *P. aeruginosa* in the biofilm layers using the LIVE/DEAD fluorescence assay was also assessed. The mixture of two fluorescent dyes, the dye SYTO-9, and the propidium iodide (included in the FilmTracer™ LIVE/DEAD Biofilm Viability Kit) was used to distinguish live and dead cells. The SYTO-9 can penetrate most membranes freely and is used for assessing total cell counts. In contrast, propidium iodide penetrates cells with compromised membranes, differentiating the damaged bacterial cells from a metabolic active, live cells. As a result, microorganisms with intact cell membranes stain fluorescent green, whereas microorganisms with damaged membranes stain fluorescent red. Analysis of representative images from an epifluorescence microscope (Figure 9) demonstrates that the Mn(II) complexes lead to morphological changes in the structure of the *P. aeruginosa* PAO1 biofilm. The presence of compounds in the culture caused the aggregation of cells into microcolonies, which was not observed in the control. This observation proves that bacteria were trying to protect themselves against the effect of the complexes tested. In addition, the tests revealed damage to the bacterial cell walls following treatment with the manganese complexes, in particular with compounds Mn-pyOH-NO_3_, [Mn-pyOH-SO_4_]_n_ and Mn-imCHO-NO_3_. These results are in accordance with the data obtained by crystal violet staining. 

Bacterial biofilms are abundant in nature, and their formation takes place in multiple stages [3]. Inhibition of biofilm formation by Mn(II) complexes may take place in two stages: (i) When planktonic bacteria adhere to a surface and initiate the formation of a microcolony; (ii) when the metabolism of a community encased in an extracellular matrix is disturbed. 

#### 2.5.3. The Effect of Mn(II) Complexes on Pyoverdine Production 

**Figure 10:**
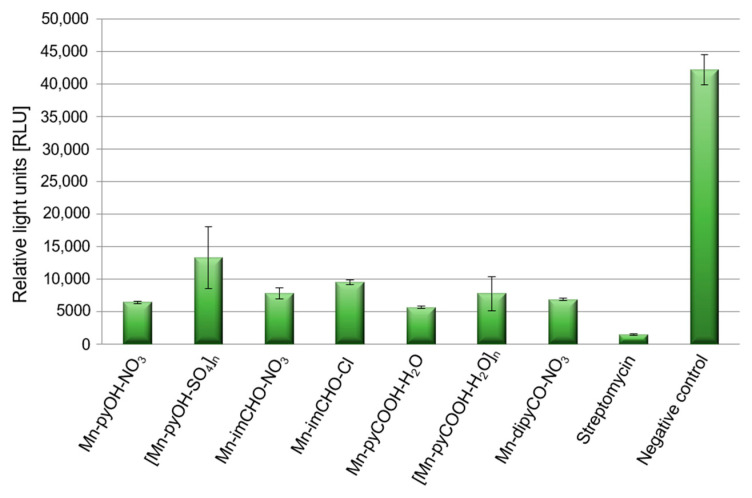
Level of pyoverdine secretion by *P. aeruginosa* strains after incubation with the manganese complex, and streptomycin as a positive control, with an untreated sample as the negative control.

Pathogenicity of *P. aeruginosa* in treatment of infection is closely related to the number of virulence factors possessed by this strain. The bacterial cell surface components (polysaccharide capsule, bacterial flagellum) and some secretory products are important virulence factors of *P. aeruginosa*, one of which is pyoverdine. Pyoverdine is a fluorescent siderophore (yellow-green) with the dihydroxyquinoline core modified by the addition of amino acid chains. It contributes in several fashions to general virulence, including regulating the production of itself, exotoxin A, and the protease PrpL. Generally, its secretion to environment is positively correlated with biofilm formation. In the experiment the spectrofluorometric method was used, and data were expressed as the relative light units (RLU). This assay demonstrates an effect of Mn(II) complexes on pyoverdine secretion (Figure 10). The Mn(II) compounds were able to decrease the production of pyoverdine in PAO1 by 68–87% in comparison to the negative control (TSB medium with inoculated bacteria). This was quite a good result considering that the presence of streptomycin reduced pyoverdine production by 96%. *P. aeruginosa* PAO1 produced the least amount of pyoverdine in the presence of Mn-pyCOOH-H_2_O (87%) compared to the other complexes. Much less pyoverdine was produced by the bacteria in the presence of [Mn-pyOH-SO_4_]_n_ (68%).

### 2.6. Cytotoxicity Activity

The cytotoxicity test of the manganese complexes against primary human fibroblasts (VH10) was performed with the MTT test. The studies have shown that the manganese complexes are not cytotoxic to primary human fibroblasts (IC_50_ higher or equal 1000 µM) (Table S6).

Digital holographic imaging (DHI) is an innovative and useful screening tool for metabolic activity investigation of human fibroblasts in the presence of metal complexes. DHI uses phase-shift imaging combined with computer algorithms to construct holographic images and enable the simultaneous study of multiple cellular parameters such as cell count, confluence, optical thickness, cell optical volume, and cell diameter [56,57]. The DHI platform HoloMonitorM4 allowed us to evaluate the effects of the Mn(II) complexes examined on cell viability of the human fibroblast as a model. In our experiment, the parameters such as a cell count and confluence were taken into account. 

**Figure 11:**
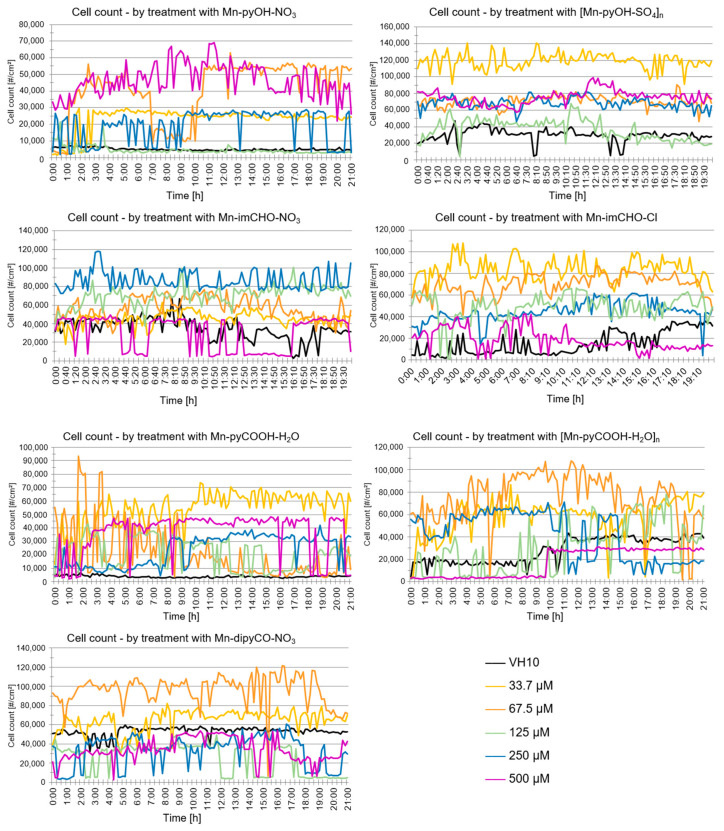
Analysis of VH10 cell count over time following the Mn(II) complexes treatment acquired using the HoloMonitorM4.

The analysis of the data indicates that the VH10 cell count did not change over time generally (20 h exposition) for all of the tested complexes (Figure 11). Nevertheless, a temporary drop in the cell count over time may have been caused by the migration of cells outside the field of view (statistically a balance is maintained). A similar effect was observed with regard to the confluence parameter over time. This parameter does not change statistically. The DHI analysis suggests that the compounds do not exhibit cytotoxicity against the human fibroblasts at the tested concentrations. The results obtained correlate well with the study gained by the MTT method.

### 2.7. Effect of Mn(II) Complexes on Catalase Activity

The series of manganese complexes presented in this paper were previously tested by our team for their mimetic properties to a manganese catalase. The preliminary studies showed that the obtained complexes in reaction with hydrogen peroxide led to quite intense gas evolution (O_2_). Moreover, it was confirmed that one of the complexes discussed here ([Mn-pyOH-SO_4_]_n_) acts as a catalyst for the H_2_O_2_ disproportionation reaction [31]. Therefore, our goal was to investigate how the presence of Mn(II) complexes affects the activity of catalase (CAT), one of the enzymes providing antioxidant protection for bacterial biofilm. CAT is the major antioxidant enzyme that defends microorganism from effects of ROS. CAT is the enzyme involved in the reduction of H_2_O_2_ via the Fenton reaction. 

**Figure 12:**
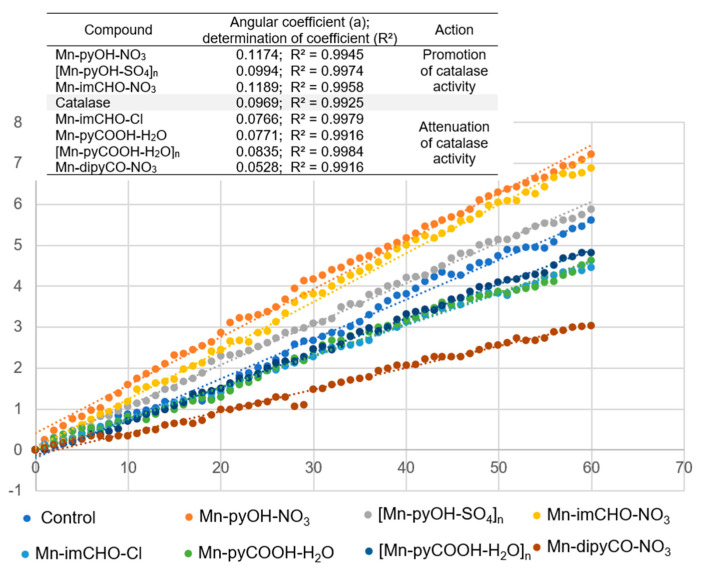
The effect of the Mn(II) complexes on the catalase activity.

First, the complexes were incubated with the catalase in the presence of a phosphate buffer, and then hydrogen peroxide was added thereto. To study the effect of the manganese complexes on the activity of CAT, its performance, in the presence of similar concentrations of the Mn complexes (0.5 mM), was investigated. The percentage of absorbance reduction over time was recorded at 240 nm. The positive control was the catalase sample in the absence of the Mn(II) complex. Figure 12 illustrates the effect of the Mn(II) complexes on the catalase activity. As Figure 12 shows, the addition of the Mn(II) complexes to the CAT solution reduced the CAT activity gradually. The highest angle of inclination of the straight line was observed for the positive control and for the complexes: Mn-pyOH-NO_3_, [Mn-pyOH-SO_4_]_n_, and Mn-imCHO-NO_3_.

The inhibitory strength of the Mn(II) complexes was investigated graphically as a slope of the linear dependence of the percentage absorbance reduction over time (Figure 12). The smaller the slope of the straight line, the lower the catalase activity and hence the greater the inhibition capacity. This parameter referring to the inhibitor strength decreases in the following order: Mn-pyOH-NO_3_ < Mn-imCHO-NO_3_ < [Mn-pyOH-SO_4_]_n_ < [Mn-pyCOOH-H_2_O]_n_ < Mn-pyCOOH-H_2_O < Mn-imCHO-Cl < Mn-dipyCO-NO_3_. The presented order largely coincides with the order of anti-biofilm activities of the studied complexes. The complexes—Mn-pyOH-NO_3_, [Mn-pyOH-SO_4_]_n_, and Mn-imCHO-NO_3_—seem to support the activity of CAT, and yet they contribute to the reduction of biofilm formation. The remaining complexes inhibited the activity of catalase and the formation of bacterial biofilm but to a much lesser extent. We deduce that the first group of the complexes competes with catalase for an active site in the catalytic center. From the literature studies, it has been found out that a manganese ion indicates some inhibitory effects on the CAT activity. This implies that manganese may competitively bind to near the heme group and be involved in the enzyme reaction [58]. On the other hand, it cannot be excluded that the complexes investigated might act as structural inhibitors through intermolecular interactions with the catalase (see HS analysis).

### 2.8. Structure–Activity (Anti-Biofilm Activity) Relationship

**Scheme 1:**
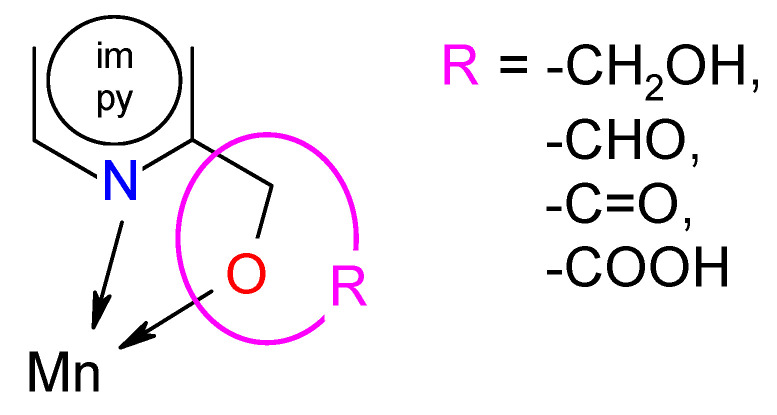
Modification in the series of the Mn(II) complexes.

The analysis of SAR enabled the determination of the chemical groups responsible for evoking a biological effect in the organism. This allowed for modification of the effect or improvement of the potency of a bioactive compound by changing its chemical structure [59]. Our group used the techniques of chemical synthesis to introduce various chemical groups into the compound and tested the modifications for their anti-biofilm effects. A series of the Mn(II) complexes with ligands consisted of the privileged structures such as imidazole (im) or pyridine (py) rings substituted different functional groups (-OH, -CHO, -C=O, -COOH) were examined (Scheme 1). A large number of heteroaromatic compounds containing pyridine or imidazole core are associated with diverse pharmacological properties such as antimicrobial, anticonvulsant, antiviral, antifungal, etc. [25,26,27,28,60].

The tuning of the activity of the imidazole and pyridine derivatives consisted of placing appropriate functional groups in the mentioned cores. Among the Mn(II) complexes with the py derivatives, the complexes with -OH groups in the ligand (Mn-pyOH-NO_3_ and [Mn-pyOH-SO_4_]_n_) were characterized by the highest activity. The influence of this substituent on biological activity was also confirmed by the literature data included in [20]. The introduction of a carboxylic group to the py ring (Mn-pyCOOH-H_2_O and [Mn-pyCOOH-H_2_O]_n_) resulted in a further activity decrease of about 10% (in the concentration of 0.5 mM). The most pronounced increase in biofilm mass (the smallest inhibition biofilm formation) occurred in the presence of the complex with -C=O group in the py ring. However, the use of the im ring as a ligand gave an unexpected effect. The Mn(II) complex with imCHO both began and ended the anti-biofilm activity row mentioned above (Anti-Biofilm Activity Section). It seems that in this group of the Mn(II) complexes, the influence of the counter-ion on biological activity was mainly observed. The introduction of nitrate(V) ions (Mn-imCHO-NO_3_) instead of chloride ions (Mn-imCHO-Cl) into the structure of the complex caused a significant increase in the biofilm mass reduction from 36% to 56%. One can also look for a relationship between the activity and the coordination number of the complexes. In contrast to the Mn(II) complexes with the im derivative, an inverse relationship is applied to the pyOH complexes in which the organic ligand has a significant influence on the activity of the complexes. Placing a nitrate(V) or sulphate(VI) ion is associated with maintaining the inhibition of biofilm at a similar level. The relationship between the structural effect and biological activity is certainly noteworthy (Table 3). The best activity is attributed to the Mn(II) complexes in which the value of a chelate angle (**∠** N-Mn-O) was exactly in the range of 71.4–72.4°. According to the analysis performed in this research and based on Table 3, the manganese compounds with a chelate angle of less than 71° had the lowest anti-biofilm activity. Meanwhile, those with a chelate angle exceeding the value of 74° inhibited *P. aeruginosa* PAO1 biofilm formation moderately. 

### 2.9. Regularity between Electrochemical Properties and Anti-Biofilm Activity

The usefulness of electrochemical techniques in the analysis of new materials should be emphasized in favor of bioactive properties determination (mainly in determining the action mechanism for antimicrobial and antitumor activities). Electrochemical parameters do not always give an absolute correlation with biological activity data due to the enormous complexity of the processes involved. Indeed, this kind of relationship is always a complex outcome, not usually dominated by a sole parameter. Great care should be taken when interpreting this type of dependency. Many other important factors must also be considered in in vivo potential biological activity, e.g., solubility, metabolism, membrane permeability [61]. Nevertheless, there are cases where such regularity is identifiable.

**Figure 13:**
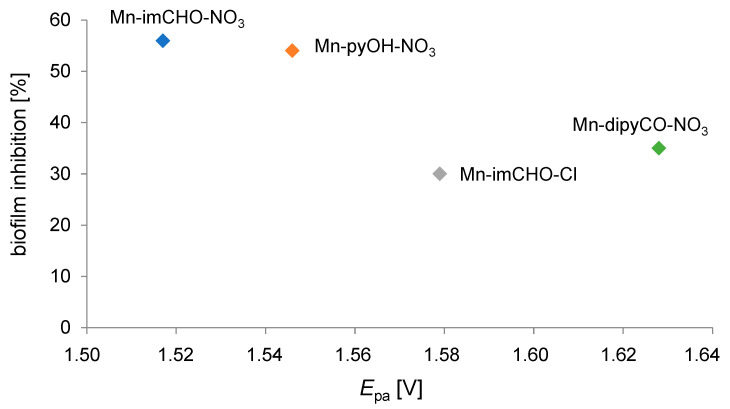
Correlation between the oxidation potential (*E*_pa_) for the Mn(II)/Mn(III) couple and the percentage biofilm inhibition against *P. aeruginosa* PAO1 strain.

Noteworthy examples are where electrochemistry, dealing with different aspects of electron transfer (ET), contributes significantly to biomedical chemistry [62]. Many of the most important physiological processes are based on redox chains, including numerous enzyme-catalyzed processes (respiratory tract). Thus, there is a set of similarities between electrochemical and biological reactions concerning electron transfer (ET) pathways [63]. In our research, we attempted to find out the relationship between the percentage value of biofilm mass inhibition and the value of the Mn(II)/Mn(III) system potential for the studied Mn complexes (Mn-pyOH-NO_3_, Mn-imCHO-NO_3_, Mn-imCHO-Cl, Mn-dipyCO-NO_3_). The results of our study indicate that the higher the half-wave potential, the higher the inhibition of the manganese complexes (Figure 13). Only in the case of the Mn-dipyCO-NO_3_ was the regularity not exactly maintained because, in the studied potential ranges, the ligand also participated in the redox reaction. The existence of such regularity can suggest the metal center can interact with biological targets based on redox reactions. Taking into account the plasticity of the redox properties of the manganese ion, it can be assumed that Mn(II) complexes may cause oxidative stress in bacterial cells.

## 3. Materials and Methods

Details of the materials, synthesis of the Mn-dipyCO-NO_3_, equipment, and physicochemical experimental procedures used are presented in the Supporting Information. The most important structural parameters of the Mn-dipyCO-NO_3_ were given in Table S7 (electronic Supplementary Material).

### 3.1. Antimicrobial Assays

The antimicrobial activities of the heteroaromatic ligands, Mn(II) complexes, and commercially available drug streptomycin were tested against standard bacterial strains: *Staphylococcus aureus* ATCC 6538P, *Escherichia coli* ATCC 8739, *Pseudomonas aeruginosa* PAO1 (biofilm model strain), and *Pseudomonas aeruginosa* LES B58 (clinical isolate). The *P. aeruginosa* PAO1 and LES B58 isolates were derived from the International *Pseudomonas aeruginosa* Reference Panel (http://bccm.belspo.be/about-us/bccm-lmg, accessed on 30 March 2021).

All bacteria species were cultivated in trypticase soy broth (TSB) medium (Biocorp, Warsaw, Poland) for 18 h at 37 °C with shaking (160 rpm). Overnight cultures of bacteria were diluted 1:100 into fresh TSB medium and then placed in each of the 96 wells of a microtiter transparent plate (Greiner, Monroe, NC, USA). The manganese(II) complexes—[Mn-pyOH-SO_4_]_n_, Mn-imCHO-NO_3_, Mn-imCHO-Cl, Mn-dipyCO-NO_3_—and the ligands—pyOH, pyCOOH—were prepared by dissolving compounds in distilled water. The compounds Mn-pyOH-NO_3_, Mn-pyCOOH-H_2_O, [Mn-pyCOOH-H_2_O]_n_, im-HO, and dipyCO were dissolved in distilled water with addition dimethyl sulfoxide (2%), which was previously tested for antibacterial activity against all test bacteria and found to have no antibacterial activity. The starting stock solution was of 2-mM concentration. 

The quantitative assay of the antibacterial activity was performed by the broth microdilution method, in 96-well microtiter plates, in order to establish the minimum inhibitory concentration (MIC). To this purpose, serial two-fold dilutions of the compounds ranging between 1 and 0.0625 mM were made. The details of the experiment were presented in our previous paper [64]. MIC values were recorded as the lowest concentration of the tested compound that completely inhibited bacterial growth, using the Infinite M200 PRO microplate reader (Tecan, Männedorf, Switzerland).

To evaluate the effect of the compounds on *P. aeruginosa* biofilm formation, a crystal violet staining assay was used, following previously described protocols [64,65]. The concentration of the investigated compounds was in the range 0.0625–1 mM. The amount of biofilm formed was determined by measuring the absorbance at λ = 595 nm with the Infinite M200 PRO microplate reader (Tecan, Männedorf, Switzerland). The measurement results, expressed in absorbance units, were converted into percentages to allow the comparison of numerical data obtained in different experiments.

Fluorescence microscopy was used to image live/dead cells in the *P. aeruginosa* PAO1 biofilm. First, the overnight culture of bacteria was diluted with fresh TSB medium (1:100). Then, the 22 × 22 mm microscope glass coverslips (Menzel-Gläser, Brunswick, Germany) were immersed in Falcon tubes (50 mL; Greiner Bio-One, Kremsmünster, Austria) containing of inoculated TSB medium supplemented with solutions of the manganese complexes (concentration: 0.5 mM). The mixture was incubated without shaking for 24 h at 37 °C. After incubation, the coverslips were carefully washed with distilled water and stained with a Filmtracer™ LIVE/DEAD™ Biofilm Viability kit (Invitrogen, Carlsbad, CA, USA) in accordance with the manufacturer’s protocol. Images were collected with an Axio Scope.A1 epifluorescence microscope (Carl Zeiss, Jena, Germany). 

The negative control (culture medium inoculated with bacteria) and positive control (antibiotic control—streptomycin) were used as references in each of the tests. All biological experiments were conducted in triplicate. 

### 3.2. Pyoverdine Production by Pseudomonas aeruginosa

Pyoverdine determination was performer in TSB medium. Measurement of pyoverdine fluorescence was performed at the excitation wavelength λ = 398 nm and the emission wavelength λ = 455 nm as described previously [66]. Experiments were carried out in triplicate in 96-well black flat-bottomed microtiter plates (Greiner, Monroe, NC, USA) for 24 h in cell-free culture medium using an Infinite M200PRO (Tecan, Männedorf, Switzerland). The assay was performed with the series of Mn(II) complexes (0.5 mM) and streptomycin as a positive control, and TSB medium with inoculated bacteria as a negative control. The siderophore secretion assay was conducted as three independent repeats. The results obtained were not disturbed by the emission band characteristic of the Mn(II) complexes.

### 3.3. Cytotoxicity Activity (MTT Test)

Primary human fibroblasts (VH10) were cultured at 37 °C in a humidified 5% CO_2_ atmosphere in plastic dishes in Dulbecco’s modified eagle medium (DMEM) supplemented with 10% heat inactivated fetal calf serum, 2 mM of l-glutamine, and antibiotics (100 units/mL penicillin and 100 µg/mL streptomycin). Cytotoxic properties of manganese complexes were measured by EZcount^TM^ MTT cell assay kit (HiMedia Laboratories Pvt., Ltd., Mumbai, India) in accordance with the manufacturer’s instructions. Cells were seeded into a 96-well plate and incubated with manganese complexes in the concentration range of 15–1000 µM for 24 h at 37 °C in a humidified atmosphere of 5% CO_2_. After incubation, a solution of MTT (3-[4,5-dimethylthiazol-2-yl]-2, 5-diphenyl tetrazolium bromide) at a concentration (1 mg/mL) was added to each well. After incubation at 37 °C for 4 h, 100 µL of 0.04 M HCl in isopropanol was added to each well and mixed thoroughly to dissolve the dark blue crystals. The measurement of absorbance of the solution related to the number of live cells was conducted on a TECAN Spark Microplate Reader (TECAN, Männedorf, Switzerland) at 570 nm with a reference wavelength 600 nm. All samples were tested in three independent experiments. Results were normalized to the control [67].

Inhibitory concentrations (IC_50_) represent the concentrations of the tested samples required to inhibit 50% cell proliferation and were calculated from the mean values of data from wells.

### 3.4. Cytotoxicity Activity (DHI Assay)

The manganese(II) complexes’ cytotoxicity was assessed by digital holographic imaging (DHI), a noninvasive, live cell imaging technique. The fibroblasts (VH10, primary human foreskin fibroblasts, CVCL_RW72) were treated with the Mn complexes at concentrations ranging from 33.7 to 500 µM, incubated, and detected by the Holomonitor M4 (Phase Holographic Imaging AB, Lund, Sweden), a phase-contrast microscope with a digital holographic function. The cells were seeded 24 h before the treatment with about 5% confluence in 1.8 mL/well of medium. The VH10 cells were cultured in Dulbecco’s modified eagle medium (DMEM, Sigma–Aldrich, Saint Louis, MO, USA) supplemented with 10% fetal bovine serum (FBS) and penicillin–streptomycin (Sigma–Aldrich, Saint Louis, MO, USA) at a concentration of 10 mL/L. For all experiments, the VH10 cells were seeded in 24-well culture plates (Lumox^®^ 24-multiwell plate (cat. 94.6000.014), Sarstedt, Numbrecht, Germany) in combination with HoloLids (Phase Holographic Imaging AB, Lund, Sweden), and placed in incubator. During the experiments, the Holomonitor was inside a cell culture incubator in a water-saturated atmosphere at 37 °C in 5% CO_2_. The cells were set to be imaged with a 10-min interval for 20 h. Three independent experiments per cell line were performed. 

### 3.5. Catalase Activity Study in the Presence of the Mn(II) Complexes

The effect of the manganese complexes on the activity of catalase (CAT) was determined using the spectrophotometric method. The decrease in absorbance due to hydrogen peroxide depletion was recorded at 240 nm by the V-780 UV-Vis/NIR spectrophotometer (Jasco, Easton, MD, USA). The tests were performed in a 50-mM potassium phosphate buffer (PBS) at 37 °C, pH = 7.0. The catalase from the bovine liver solution (Sigma–Aldrich, St. Louis, MO, USA, lyophilized powder, ≥10,000 units/mg protein) was prepared in the PBS buffer as an initial dilution of ~1000 units/mL at 37 °C. The hydrogen peroxide solution, approximately 0.03% (*w*/*w*), was prepared by dilution in the PBS buffer to achieve the absorbance close to 1 a.u, and the PBS was used as a blank. The manganese complexes were diluted in the PBS buffer to stock the concentration of 1 mM. The mixture of 500 µL of the hydrogen peroxide, 1 µL of the catalase (final 1U of catalase activity), and 500 µL of the manganese complexes, previously incubated in a water bath at 37 °C, was added to a quartz cuvette (10 mm, 1 mL), closed by a cover, and placed in the spectrophotometer. The absorbance readings were performed in 10-s intervals during the first minute of the incubation time at 37 °C. The results were presented as the dependence on the percentage of the absorbance reduction over time. 

### 3.6. Statistical Analysis

Statistical analysis was performed using one-way analysis of variance (ANOVA). Significance was set at *p* < 0.05.

## 4. Conclusions

The series of manganese(II) complexes was evaluated in the context of a biotechnological application. For the purposes of the aforementioned application, a new Mn(II) complex (Mn-dipyCO-NO_3_) was synthesized and characterized by the crystal structure as well as by spectroscopic, thermal, and magnetic methods. The strength of the O atom ligation to the Mn(II) in the Mn-dipyCO-NO_3_ is smaller than for the other functional groups of py studied in this paper. Among the complexes considered, the hydroxyl group of py has the highest affinity for the Mn(II) ion, and next, the carboxylic group of py. The detailed HS analysis indicated that some types of intermolecular interactions in the crystals of the Mn(II) complexes can possibly affect anti-biofilm activity. Various functional groups in the ligand structure can produce the various possibility of interactions with biological targets in the bacterial biofilm. As expected, modification in the structure of the Mn(II) complexes also affected the anti-biofilm activity. The activity row for the py complexes is as followed: pyOH > pyCOOH > pyCO. However, for complexes with imCHO, the type of coordinated inorganic ion is significant. The inhibitory effect of the Mn(II) complexes on *P. aeruginosa* biofilm was found to be non-specific, and it was observed at the concentration range of 0.25–0.5 mM only (targeted action). The evaluated complexes inhibited the growth of both the planktonic form of *P. aeruginosa* and biofilm. However, the inhibition of biofilm formation in the presence of the Mn(II) complexes was significantly higher than the inhibition of planktonic cells of bacteria. This result correlates well with the images obtained by epifluorescence microscopy. Furthermore, the pyoverdine secretion assay demonstrates that Mn(II) complexes effect on pyoverdine production causing its significant decrease. The cytotoxicity studies, which are of paramount relevance to possible applicability, indicated the non-toxic effect of the Mn(II) complexes on primary human fibroblasts. For this purpose, the MTT method and an innovative DHI platform HoloMonitorM4 were used. It is worth noting the electrochemical studies, which show the varied oxidation efficiency of the manganese(II) complexes and can adequately reflect their biological activity. Interestingly, the regularity between the electrochemical and anti-biofilm studies is observed. It suggests that the metal center can interact with biological targets based on redox reactions. Taking into account the properties determined, it can be assumed that the Mn(II) complexes may contribute to oxidative stress by disturbance of antioxidant protection for bacterial biofilm. Indeed, the studies of the effect of the complexes on CAT activity demonstrate that the Mn-imCHO-Cl, Mn-pyCOOH-H_2_O, [Mn-pyCOOH-H_2_O]_n_, and Mn-dipyCO-NO_3_ complexes attenuate the catalase activity, with the exception of the Mn-pyOH-NO_3_, [Mn-pyOH-SO_4_]_n_, and Mn-imCHO-NO_3_ complexes, and the presence of which seems to promote the catalase activity. The analysis of the bioassay results suggests two probable actions of the evaluated compounds:

- The first one regards the induction of oxidative stress in bacterial cells by the inhibition effect on the CAT enzyme;
- The other one is connected with the participation of the complexes in the disturbance of adhesion of bacterial cells (supramolecular interactions).

To investigate these aspects in detail, further specific tests are essential. To summarize, the Mn(II) complexes might be a suitable candidates for the development of a new anti-biofilm agents. The complexes Mn-pyOH-NO_3_, [Mn-pyOH-SO_4_]_n_, and Mn-imCHO-NO_3_ seem to be most promising. In order to evaluate the practical potential of these compounds for biotechnological application, further studies regarding the growth inhibition mechanism should be conducted.

## Figures and Tables

**Table 1:** Thermoanalytical results (TG and DTG) of Mn-dipyCO-NO_3_.

Step	TG Range/K	DTG _max_/K	Mass Loss Obs. (Calcd.)/%	Assignment
I	448–540	483	34.37 (32.93)	0.98 dipyCO
II	540–628	583	16.58 (16.81)	2 NO_2_
III	628–1073	663	32.98 (34.38)	1.02 dipyCO
Total			83.93 (84.12)	Leaving MnO_2_ residue

**Table 2:** Structural criteria used for assigning nitrate modes for the Mn-dipyCO-NO_3_ complex [Å, °].

Criteria	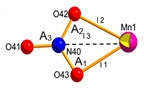	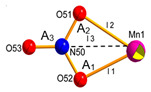	Bidentate Nitrate
l_2_-l_1_	0.07	0.14	<0.3 [34]
A_1_-A_2_	2.78	6.33	<14 [34]
l_3_-l_2_	0.37	0.33	>0.2 [34]
A_3_	176.42	174.37	>162 [34]

**Table 3:** Correlation between selected structural parameters and anti-biofilm activity for the Mn(II) complexes.

Complex	Mn-N (Ligand) [Å]	Mn-O (Ligand) [Å]	Space Group	∠ N-C-C-O [°]	∠ N-Mn-O Chelate [°]	∠ O-Mn-O Chelate [°]	CN	% Inhibit. Biofilm (0.5 mM)	% Inhibit. Biofilm (0.25 mM)
Mn-pyOH-NO_3_	2.2792(1)	2.2296(1)	*C* 2/*c*	7.31	71.36	51.36	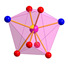	54	49
[Mn-pyOH-SO_4_]_n_	2.247(2)	2.2325(2)	*P* 2_1_/*c*	−26.23	72.37	–	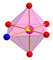	53	48
Mn-imCHO-NO_3_	2.2185(1) 2.2250(1) *2.222 (avg.)*	2.3897(1)-2.4693(1)	*P* $\bar{1}$	−2.91 −0.30	71.4 672.67 *72.07 (avg.)*	49.03 52.69 *50.86 (avg.)*	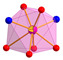	56	52
Mn-imCHO-Cl	2.2254(2) 2.2319(2) *2.229 (avg.)*	2.3955(2) 2.5951(2)	*P* $\bar{1}$	−1.14 −0.31	69.41 72.58 *71.00 (avg.)*	–	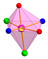	29	24
Mn-pyCOOH-H_2_O	2.2805(1)	2.1390(1)	*P* $\bar{3}$	−14.22	74.13 *74.09 (avg.)*	–	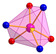	48	45
[Mn-pyCOOH-H_2_O]_n_	2.2630(4)	2.137(3), 2.167(3)	*P ca* 2_1_	2.88	74.72	–	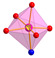	45	44
Mn-dipyCO-NO_3_	2.2715(2) 2.3180(2) *2.295(avg.)*	2.3144(2) 2.3049(2)	*P* $\bar{1}$	−3.14 −3.21	69.25 69.03 *69.14 (avg.)*	54.34 55.19 *54.77 (avg.)*	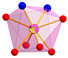	36	21

## Data Availability

Data sharing not applicable.

## Supplementary Materials

The following are available online at https://www.mdpi.com/article/10.3390/ijms22094847/s1, Figure S1: TG and DTG curves showing the thermal behavior of Mn-dipyCO-NO_3_, Figure S2: The 2D full fingerprint plots, Figure S3: Cyclic voltammogram of the Mn-pyOH-NO_3_ recorded at scan rates from 4.20 to 50 mV s^−1^ in CH_3_CN solution containing 0.1 M TBAPF_6_ (CV conditions: BDDE, Ø = 3 mm, T = 25 °C), Figure S4: The CV (**a**) and DPV (**b**) curves recorded in CH_3_CN/glacial CH_3_COOH solution containing 0.1 M of TBAPF_6_ and 1 mM solution of dipyCO, (CV conditions: BDDE, Ø = 3 mm, scan rate 100 mV s^−1^, T = 25 °C; DPV conditions: BDDE, Ø = 3 mm, pulse amplitude 20 mV, pulse width 60 ms, scan rate 20 mV s^−1^), Figure S5: The CV curves recorded in CH_3_CN/glacial CH_3_COOH solution containing 0.1 M of TBAPF_6_ and 1 mM of solution of the Mn(NO_3_)_2_ (CV conditions: BDDE, Ø = 3 mm, scan rates from 4.20 mV s^−1^ to 50 mV s^−1^, T = 25 °C), Table S1: The characteristic FT-IR absorption frequencies (cm^−1^) of the ligand (dipyCO) and the Mn-dipyCO-NO_3_, Table S2: Selected bond lengths [Å] and valence angles [°] for Mn-dipyCO-NO_3_, Table S3: Hydrogen bonds for the Mn-dipyCO-NO_3_ complex [Å] and [°], Table S4: Electrochemical data [in V vs. Ag/AgCl (3M KCl)] of the manganese(II) complexes obtained by cyclic voltammetry, Table S5: Bacteriostatic activities of the investigated Mn(II) complexes, Mn(II) salts, and ligands as MIC concentrations, expressed in mM and μg/mL, Table S6: IC_50_ values of the investigated Mn(II) complexes against VH10 cells, Table S7: Crystal data and structure refinements for the Mn-dipyCO-NO_3_.
