# Supplementary material for: Tuning Anti-Biofilm Activity of Manganese(II) Complexes: Linking Biological Effectiveness of Heteroaromatic Complexes of Alcohol, Aldehyde, Ketone, and Carboxylic Acid with Structural Effects and Redox Activity
Source: Int J Mol Sci. 2021 May 3;22(9):4847. doi: 10.3390/ijms22094847 (PMC8124774; doi:10.3390/ijms22094847)
Supplement: Supplementary file 1 [file ijms-22-04847-s001.zip › ijms-1184741-supplementary.pdf]

Electronic Supplementary Information

Tuning Anti-biofilm Activity of Manganese(II) Complexes: Linking Biological Effectiveness of Heteroaromatic Complexes of Alcohol, Aldehyde, Ketone and Carboxylic Acid with Structural Effects and Redox Activity

Agnieszka Jabłońska-Wawrzycka*[1], Patrycja Rogala[1], Grzegorz Czerwonka[2], Sławomir Michałkiewicz[1], Maciej Hodorowicz[3], Katarzyna Gałczyńska[2], Beata Cieślak[4], Paweł Kowalczyk[5]

[1] *Institute of Chemistry, Jan Kochanowski University of Kielce, 7 Uniwersytecka Str., 25-406 Kielce, Poland,*
e-mail: agajw@yahoo.com; agnieszka.jablonska-wawrzycka@ujk.edu.pl
[2] *Institute of Biology, Jan Kochanowski University of Kielce, 7 Uniwersytecka Str., 25-406 Kielce, Poland,*
[3] *Faculty of Chemistry, Jagiellonian University, 2 Gronostajowa Str., 30-387 Kraków, Poland*
[4] *Warsaw College of Engineering and Health, 18 Bitwy Warszawskiej 1920r. Str., 02-366 Warszawa, Poland;*
[5] *Department of Animal Nutrition, The Kielanowski Institute of Animal Physiology and Nutrition, Polish Academy of Sciences, 3 Instytucka Str., 05-110 Jabłonna, Poland*

CONTENTS

1. Experimental section……………………………………………...S2 – S5

2. Supporting figures……………………………………….......S6 – S9

3. Supporting tables…………………………………….........S10 – S13

4. Supporting references…………………………………………….S14

1. Experimental section

1.1. Materials

Chemical reagents were purchased from commercial sources and were used without further purification: $Mn(NO_3)_2{\cdot}4H_2O$ (Merc), dipyridin-2-ylmethanone (Sigma Aldrich, 99%). All solvents used in this work were of analytical grade and were used as received. Manganese(II) complexes: Mn-pyOH-NO_3, [Mn-pyOH-SO_4]$_n$, Mn-imCHO-NO_3, Mn-imCHO-Cl, Mn-pyCOOH-H_2O and [Mn-pyCOOH-H_2O]$_n$ (where pyOH – 2-hydroxymethylpyridyne, imCHO – 5(4)-carbaldehyde-4(5)-methylimidazole, pyCOOH – pyridine-2,3-dicarboxylic anion) were synthesized according to the previously described procedures [1–5]. The obtained Mn(II) complexes were characterised by spectroscopic and thermal methods, magnetic measurements, and crystal structures were confirmed.

1.2. Synthesis of Mn-dipyCO-NO_3

To prepare the manganese(II) complex a procedure was followed: dipyridin-2-ylmethanone (2 mmol, 0.2964g) was dissolved in CH_3CN (25 ml) and the mixture was stirred. A solution of manganese(II) nitrate(V) (1 mmol, 0.251g) dissolved in the same solvent (25 ml) was added, dropwise, to the ligand solution. Obtained yellow solution was then stirred for 2h and allowed in room temperature to evaporate the solvent. The orange fine-crystalline product was collected by filtration and washing with CH_2Cl_2 and then dried *in vacuo*. The pure crystallized product was obtained from acetonitrile. The product was collected in 36.7% yield. Melting point: 195°C. Elemental analysis (%), Calc. for $MnC_{22}H_{16}N_6O_8$: C 48.28, H 2.95, N 15.35; Found: C 48.29, H 2.83, N 15.47; FT-IR (cm^{-1}): 3502(vs), 3113(w), 3072(w), 1653(s), 1643(s), 1587(s), 1568(s), 1448(vs), 1433(vs), 1331(s), 1312(vs), 1298(vs), 1237(vs), 1176(s), 1156(s), 1103(s), 1051(s), 1034(s), 1009(s), 995(vs), 955(vs), 837(s), 818(s), 752(vs), 735(s) 670(vs), 636(vs), 619(vs).

1.3. Physical measurements

The elemental analysis (C, H and N) was carried out using a Vario Micro Cube Analyser (Elementar). The IR spectra were recorded on a Nicolet 380 FT-IR spectrophotometer (Thermo Scientific) in the spectral range 4000 – 500 cm^{-1} using the ATR-diffusive reflection method. Magnetic susceptibility of polycrystalline sample was investigated at 70 - 300K. The measurement was caried out using the Gouy’s method. Mass changes were obtained from Cahn-Ventron RM-2 electrobalance. The calibrant employed was $Hg[Co(SCN)_4]$ for which the magnetic susceptibility of $8.08 \cdot 10^{-3} cm^3\ mol^{-1}$. Corrections for diamagnetism of the constituent atoms was calculated by the use of Pascal's constants [6,7].

Magnetic moment at room temperature was calculated according to the equation: μ_{eff} = $2.828 \cdot \sqrt{\chi_A \cdot \mathrm{T}}$, $\chi_A = \chi_M + \mathrm{DIA}$

The thermal analysis (TG/DTG) was carried out by using a TG/SDTA 851^e Mettler-Toledo thermobalance. The experiment was performed under the dynamic nitrogen atmosphere at a heating rate of 5°C/min in the temperature range of 298 – 1073 K using an α-Al_2O_3 crucible. The thermoanalytical curves were obtained using STAR^e System Mettler-Toledo software. Voltammetric experiments were performed using a Model M161 electrochemical analyser (mtm-anko, Poland) and controlled *via* a Pentium computer using EALab 2.1 software. In a three-electrode cell, a boron-doped diamond electrode (BDDE) of 3 mm diameter, A = 0.0707 cm^2 (BioLogic), and platinum wire were used as a working electrode and a counter electrode, respectively. All potentials were measured against the external silver chloride reference electrode (Ag/AgCl) with 3 M KCl solution. To avoid water leakage and chloride ions, the reference electrode was isolated from the solution by a salt bridge with a frit of Victor Glass. Electrochemical measurements were carried out at room temperature (25 ± 1°C) and at different scan rates of potential (from 4.2 to 50 mVs^{-1}). Electrochemical investigations of manganese(II) complexes and free ligands were performed in an acetonitrile or acetonitrile/glacial acetic acid mixture (4 : 1, *v/v*) (Chempur, Merc) containing 1 mM compound with 0.1 M tetrabutylammonium hexafluorophosphate ($TBAPF_6$) (Fluka, electrochemical grade) as a supporting electrolyte. Some experiments were performed with the use of differential pulse voltammetry (DPV) on the same BDDE, as a working electrode. DPV voltammograms were registered using a pulse amplitude of 20 mV, pulse width of 60 ms and scan rate of 20 mV s^{-1}. To verify the reversibility of the redox couples and the number of electrons exchanged, CV diagnostic criteria were applied. The theoretically value expected for a reversible one electron exchange process is accorded to equation E_p-$E_{p/2}$ = 0.0565 V (where E_p denotes the potential of the anodic peak, $E_{p/2}$ - the potential at the half-height of the peak current) [8]. For the irreversible process, value exceeds the theoretically value for E_p-$E_{p/2}$ is assumed. Determination of process reversibility allows to estimate of exchange electron number. For the reversible system E_p-$E_{p/2}$ = 0.0565/n V equation is used. In the case of irreversible processes, the criterium E_p-$E_{p/2}$ = 0.0477/nα is satisfied (n - the number of electrons exchanged, α - the transition coefficient is most often 0.3) [8].

1.4. Crystal structure determination and refinement (XRD)

Diffraction intensity data for a single-crystal of Mn-dipyCO-NO_3 was collected at 100 K on a KappaCCD (Nonius) diffractometer with graphite-monochromated MoKα radiation (λ = 0.71073 Å) and processed using the DENZO/SCALEPACK program package [9,10]. Corrections for

Lorentz, polarization and absorption effects were applied. The structure was solved by direct methods using the SHELXL-97 program package [11] and refined using a full-matrix least square procedure on F^2 using SHELXL-2017/1 [12,13]. Anisotropic displacement parameters for all non-hydrogen atoms and isotropic temperature factors for hydrogen atoms were introduced. The H atoms were generated geometrically and refined using a riding model. Molecular drawings were obtained using DIAMOND [14] software. Further details of the structure analysis is given in Table S7.

Crystallographic data for the structure reported here have been deposited with the Cambridge Crystallographic Data Centre, as supplementary publication number CCDC 2059976. These data can be obtained free of charge *via* https://www.ccdc.cam.ac.uk/structures/ (or from the CCDC, 12 Union Road, Cambridge CB2 1EZ, UK; fax: +44-1223-336033).

The XRD investigations were carried out on a DRON-2 (Russia) diffractometer connected to an IBM computer, stepwise, over the 2θ angle range 15-80°, using CuK_{α} radiation. The products of decomposition were studied using X-ray powder diffraction and Powder Diffraction File [15].

1.5. Hirshfeld surface analysis

Molecular Hirshfeld surfaces calculations were performed using the Crystal Explorer package *ver.* 3.1 [16]. When the .cif file of the title compounds was entered into the Crystal Explorer program, all of the bond lengths to hydrogen were automatically modified to the standard neutron values (CH = 1.083 A).

The Hirshfeld surface of a molecule is mapped using the descriptor d_{norm}, which encompasses two factors: d_{i}, representing the distance of any surface point nearest to the internal atoms, and d_{e}, representing the distance of the surface point nearest to the exterior atoms. The calculated d_{norm} is a normalized contact distance, which has been defined in terms of d_{i}, d_{e} and the van der Waals (vdW) radii of the atoms [17]:

$$d_{\text{norm}} = \frac{d_i - \mathrm{r}_i^{vdW}}{\mathrm{r}_i^{vdW}} + \frac{d_{\text{e}} - \mathrm{r}_e^{vdW}}{\mathrm{r}_e^{vdW}}$$

The value of d_{norm} was negative or positive when intermolecular contacts were shorter or longer than r vdW, respectively. The d_{norm} values were mapped onto the Hirshfeld surfaces using a red-blue-white colour scheme as follows: red regions represented closer contacts and a negative d_{norm} value; blue regions represented longer contacts and a positive d_{norm} value; and white regions represented the distance of contacts equal to exactly the vdW separation with a d_{norm} value of zero [17]. In turn, the *shape index* is highly sensitive to very subtle changes in the surface shape. The information delivered by the *shape index* are consistent with 2D fingerprint plots. The whole fingerprint region and all interactions are a combination of d_{e} and d_{i}. The surfaces were made to be

transparent to allow visualization of the molecular moiety in a similar orientation for all of the structures around which they were calculated. The 2D fingerprint plots were produced using the standard 0.6 – 2.5 Å and view with the d_e and d_i distance scales displayed on the graph axes. The distance from the Hirshfeld surface to the nearest nucleus inside and outside the surface has been marked by d_i and d_e, respectively.

1.6. Stability and solubility

The solubility of the complexes was tested in solvents such as water, methanol, ethanol, propan-2-ol, dimethyl sulfoxide, *N,N*-dimethylformamide, acetone and acetonitrile. The complexes were found to be well soluble in water, alcohols, acetonitrile, dimethyl sulfoxide (DMSO) and otherwise insoluble in other common organic solvents as acetone and *N,N*-dimethylformamide (DMF).

Solution stability of Mn(II) complexes was studied by spectroscopic method. Compounds Mn-pyOH-NO_3, Mn-pyCOOH-H_2O and [Mn-pyCOOH-H_2O]$_n$ were dissolved in an aqueous solution with the addition of dimethyl sulfoxide (the final concentration of DMSO did not exceed 2% by volume - analogously to biological tests), and other compounds were dissolved in distilled water. The concentrations of prepared solutions of the Mn(II) complexes were 1 mM. UV-Vis spectra of investigated compounds were recorded after dissolution, as well as after 24 h of incubation at 37°C.

2. Supporting figures

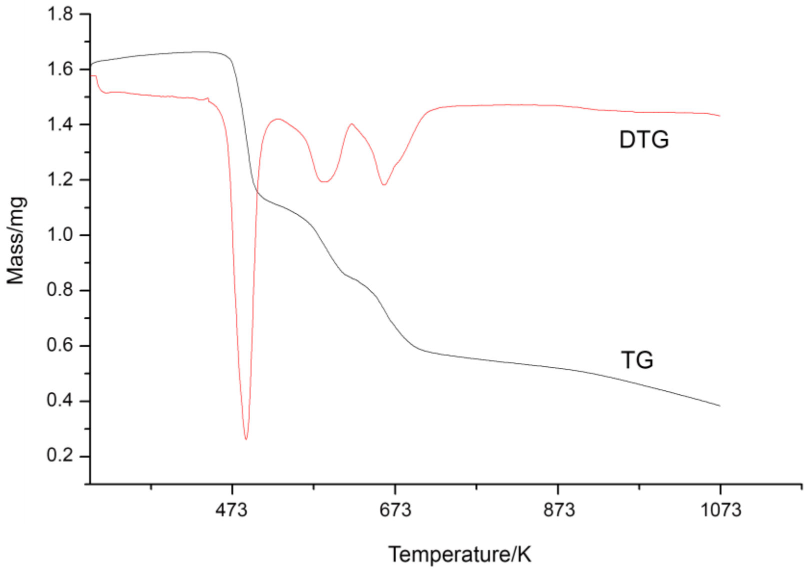

Figure S1. TG and DTG curves showing the thermal behaviour of Mn-dipyCO-NO_3.

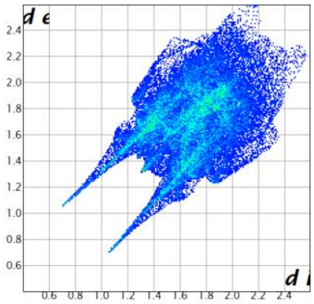

Mn-pyOH-NO_3

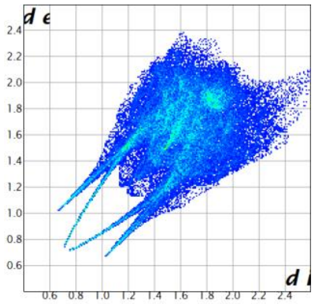

[Mn-pyOH-SO_4]$_n$

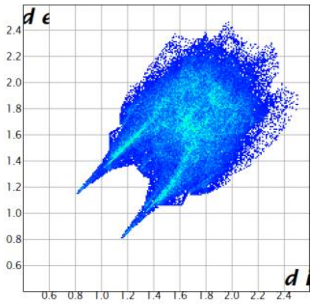

Mn-imCHO-NO_3

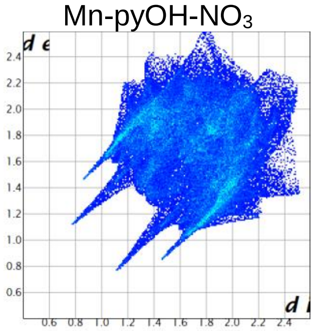

Mn-imCHO-Cl

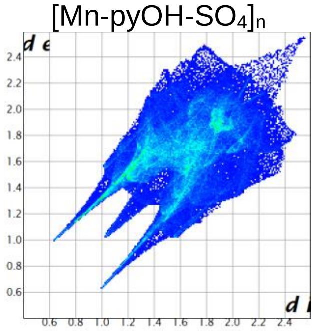

Mn-pyCOOH-H_2O A

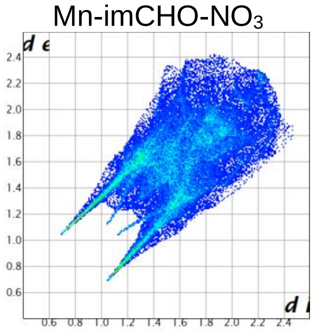

[Mn-pyCOOH-H_2O]$_n$

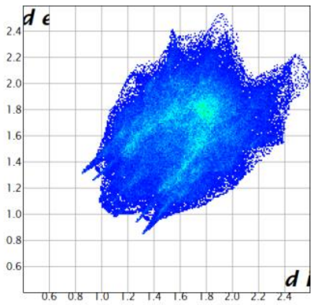

Mn-dipyCO-NO_3

Figure S2. The 2D full fingerprint plots.

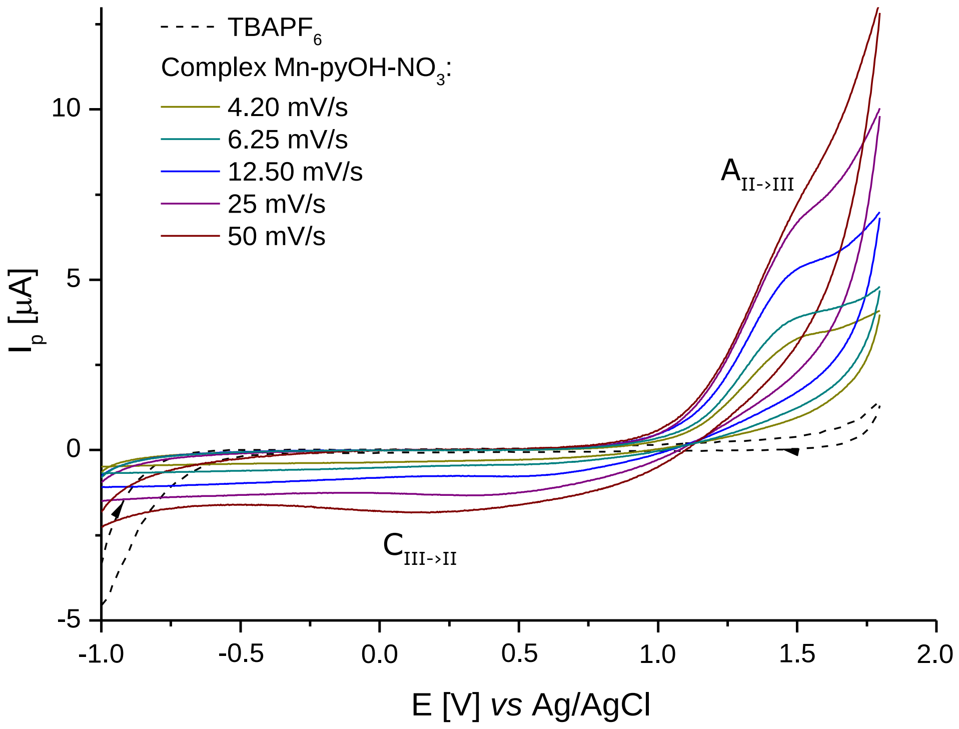

Figure S3. Cyclic voltammogram of the Mn-pyOH-NO_3 recorded at scan rates from 4.20 mV s^{-1} to 50 mV s^{-1} in CH_3CN solution containing 0.1 M $TBAPF_6$ (CV conditions: BDDE, Ø = 3 mm, T = 25°C).

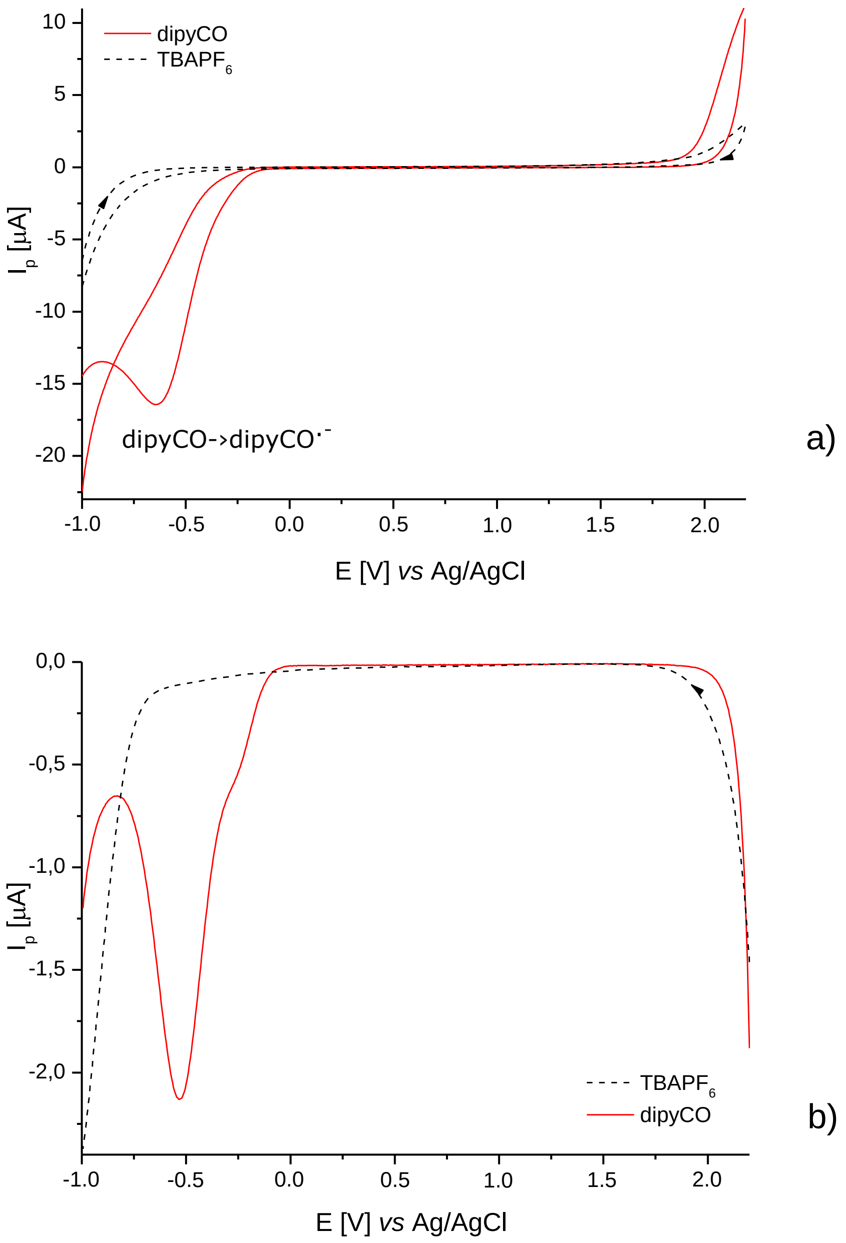

Figure S4. The CV (a) and DPV (b) curves recorded in CH_3CN/glacial CH_3COOH solution containing 0.1 M $TBAPF_6$ and 1 mM solution of dipyCO, (CV conditions: BDDE, Ø = 3 mm, scan rate 100 mV s^{-1}, T = 25°C; DPV conditions: BDDE, Ø = 3 mm, pulse amplitude 20 mV, pulse width 60 ms, scan rate 20 mV s^{-1}).

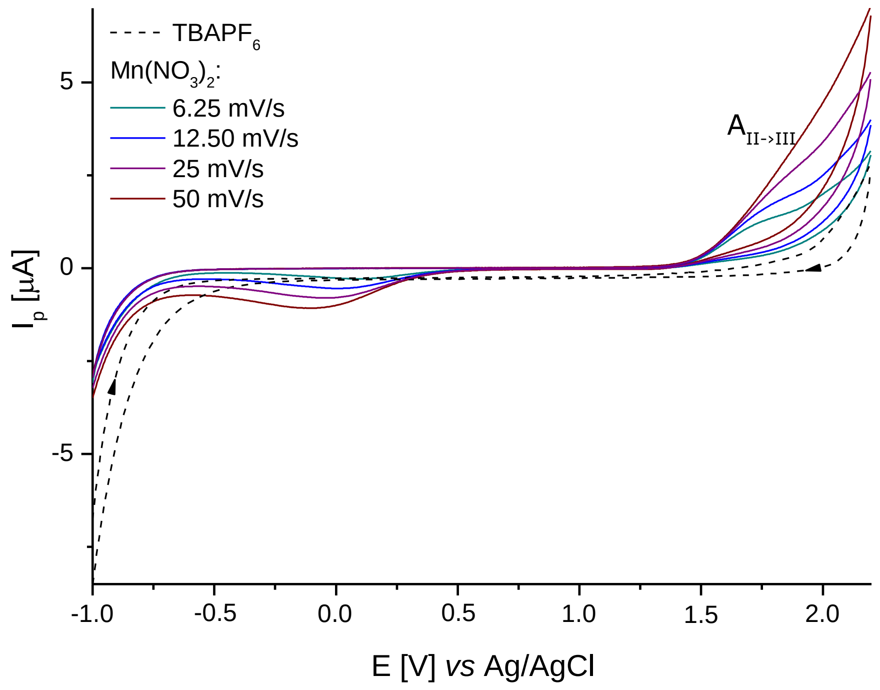

Figure S5. The CV curves recorded in CH_3CN/glacial CH_3COOH solution containing 0.1 M $TBAPF_6$ and 1 mM solution of the $Mn(NO_3)_2$ (CV conditions: BDDE, Ø = 3 mm, scan rates from 4.20 mV s^{-1} to 50 mV s^{-1}, T = 25°C).

3. Supporting tables

Table S1. The characteristic FT-IR absorption frequencies (cm^{-1}) of the ligand (dipyCO) and the Mn-dipyCO-NO_3.

Assignment	dipyCO	Mn-dipyCO-NO_3
$\nu_{C-H\ ar}$	3068, 3049	3113, 3072
$\nu_{C=O}$	1680	1653, 1643 (Δ= 27, 37)
$\nu_{C=C}$, $\nu_{C=N}$	1578, 1568	1587, 1569
breathing ring vibration	1098, 1090	1103
$\delta_{C-H\ (in\text{-}plane)}$	991, 943	995, 955
$\delta_{C-H(out\text{-}of\text{-}plane)}$	754, 741	764, 752
$\nu_{NO_3^-}$	–	1448 (ν_1)(A_1)<br>1298 (ν_4)(B_2)<br>1034 (ν_2)(A_1)<br>818 (ν_6)(B_1)<br>735(ν_5)(B_2)<br>1771, 1734 (ν_1+ ν_4) (Δ= 37)
$\nu_{Mn-O(dipyCO)}$	–	228, 219
ν_{Mn-N}	–	368, 361

Table S2. Selected bond lengths [Å] and valence angles [°] for Mn-dipyCO-NO_3.

Bond lengths (Å)			
Mn(1)-N(1)	2.3176(2)	Mn(1)-O(42)	2.3562(1)
Mn(1)-N(21)	2.2711(1)	Mn(1)-O(43)	2.2908(1)
Mn(1)-O(8)	2.3032(1)	Mn(1)-O(51)	2.4245(1)
Mn(1)-O(28)	2.3137(1)	Mn(1)-O(52)	2.2814(1)
**Valance angles (°)**			
N(21)-Mn(1)-O(52)	86.41(5)	O(28)-Mn(1)-N(1)	84.82(5)
N(21)-Mn(1)-O(43)	159.58(5)	N(21)-Mn(1)-O(42)	143.46(4)
O(52)-Mn(1)-O(43)	89.00(5)	O(52)-Mn(1)-O(42)	83.54(4)
N(21)-Mn(1)-O(8)	84.94(5)	Cl(3)-Mn(1)-Cl(1)	90.02(3)
O(52)-Mn(1)-O(8)	125.29(4)	O(43)-Mn(1)-O(42)	55.22(4)
O(43)-Mn(1)-O(8)	81.53(4)	O(8)-Mn(1)-O(42)	128.91(4)
N(21)-Mn(1)-O(28)	69.21(4)	O(28)-Mn(1)-O(42)	74.69(4)
O(52)-Mn(1)-O(28)	82.62(4)	N(1)-Mn(1)-O(42)	86.59(5)
O(43)-Mn(1)-O(28)	129.84(4)	N(21)-Mn(1)-O(51)	81.69(5)
O(8)-Mn(1)-O(28)	141.27(4)	O(52)-Mn(1)-O(51)	54.37(4)
N(21)-Mn(1)-N(1)	95.49(5)	O(43)-Mn(1)-O(51)	79.48(4)
O(52)-Mn(1)-N(1)	165.70(5)	O(8)-Mn(1)-O(51)	70.93(4)
O(43)-Mn(1)-N(1)	93.85(5)	O(28)-Mn(1)-O(51)	129.47(4)
O(8)-Mn(1)-N(1)	69.00(5)	N(1)-Mn(1)-O(51)	139.93(5)
		O(42)-Mn(1)-O(51)	118.93(4)

Table S3. Hydrogen bonds for the Mn-dipyCO-NO_3 complex [Å] and [°].

D-H···A	d(D-H)	d(H···A)	d(D···A)	<(DHA)
C(23)-H(23)···N(31)	0.95	2.24	2.885(2)	124.3
C(25)-H(25)···O(53)^{i}	0.95	2.65	3.489(2)	147.7
C(26)-H(26)···O(8)	0.95	2.52	3.101(2)	119.7
C(6)-H(6)···O(28)	0.95	2.63	3.176(3)	116.9
C(5)-H(5)···O(51)	0.95	2.32	3.219(2)	156.8
C(3A)-H(3A)···O(43)^{iii}	0.95	2.59	3.284(5)	130.0
C(3A)-H(3A)···N(11A)	0.95	2.31	2.908(7)	120.6
C(16A)-H(16A)···O(41)^{iii}	0.95	2.54	3.209(5)	127.7
C(3B)-H(3B)···N(11B)	0.95	2.31	2.911(6)	120.2
C(16B)-H(16B)···O42(3)^{iii}	0.95	2.50	3.278(5)	139.2

Symmetry transformations used to generate equivalent atoms (i) x-1,y,z; (ii) x,y+1,z; (iii) –x+2,-y+1,-z+1

Table S4. Electrochemical data [in V *vs* Ag/AgCl (3M KCl)] of the manganese(II) complexes obtained by cyclic voltammetry.

Scan rate [mVs^{-1}]	E_{pa} [V]	$E_{pa/2}$ [V]	$E_{pa} – E_{pa/2}$ [V]	nα	n (α=0.3)
**Mn-pyOH-NO_3**					
4.20	1.546	1.384	0.162	0.30	1.0
6.25	1.592	1.406	0.186	0.26	0.9
**Mn-imCHO-NO_3**					
4.20	1.517	1.362	0.155	0.31	1.0
6.25	1.559	1.380	0.179	0.27	0.9
**Mn-imCHO-Cl**					
4.20	1.579	1.404	0.175	0.27	0.9
6.25	1.608	1.413	0.195	0.25	0.8
**Mn-dipyCO-NO_3**					
4.20	1.628	1.429	0.199	0.24	0.8
6.25	1.660	1.458	0.198	0.24	0.8
**$Mn(NO_3)_2$**					
6.25	~1.8	~1.6	0.2	0.26	0.9

Conditions: 0.1 M $TBAPF_6$ in mixed solvent; CH_3CN/glacial CH_3COOH; CV: BDDE (Ø = 3 mm), scan rate of 4.20 mV s^{-1} and 6.25 mV s^{-1}.

Table S5. Bacteriostatic activities of the investigated Mn(II) complexes, Mn(II) salts and ligands as MIC concentrations, expressed in mM and μg/ml.

Compound	BACTERIA							
	S. aureus		*E. coli*		*P. aeruginosa PAO1*		*P. aeruginosa LES B58*	
	mM	μg/ml	mM	μg/ml	mM	μg/ml	mM	μg/ml
$MnCl_2·4H_2O$	> 1	> 198	> 1	> 198	> 1	> 198	> 1	> 198
$Mn(NO_3)_2·4H_2O$	> 1	> 251	> 1	> 251	> 1	> 251	> 1	> 251
$MnSO_4·H_2O$	> 1	> 169	> 1	> 169	> 1	> 169	> 1	> 169
pyOH	> 1	> 109	> 1	> 109	> 1	> 109	> 1	> 109
imCHO	> 1	> 110	> 1	> 110	> 1	> 110	> 1	> 110
pyCOOH	> 1	> 167	> 1	> 167	> 1	> 167	> 1	> 167
dipyCO	> 1	> 184	> 1	> 184	> 1	> 184	> 1	> 184
Mn-pyOH-NO_3	> 1	> 397	> 1	> 397	> 1	> 397	> 1	> 397
[Mn-pyOH-SO_4]$_n$	> 1	> 278	> 1	> 278	> 1	> 278	> 1	> 278
Mn-imCHO-NO_3	1	399	> 1	> 399	> 1	> 399	> 1	> 399
Mn-imCHO-Cl	> 1	> 346	> 1	> 346	> 1	> 346	> 1	> 346
Mn-pyCOOH-H_2O	> 1	>1270	> 1	>1270	> 1	>1270	> 1	>1270
[Mn-pyCOOH-H_2O]$_n$	> 1	> 274	> 1	> 274	1	274	> 1	> 274
Mn-dipyCO-NO_3	> 1	> 547	> 1	> 547	> 1	> 547	> 1	> 547
Streptomycin	0.125	73	0.0625	36	0.0625	36	0.125	73

pyOH – 2-hydroxymethylpyridyne, imCHO – 5(4)-carbaldehyde-4(5)-methylimidazole, pyCOOH – pyridine-2,3-dicarboxylic anion, dipyCO – dipyridin-2-ylmethanone

Table S6. IC_{50} values of the investigated Mn(II) complexes against VH10 cells.

Complex	IC_{50} (μM)
Mn-pyOH-NO_3	1000
[Mn-pyOH-SO_4]$_n$	>1000
Mn-imCHO-NO_3	>1000
Mn-imCHO-Cl	>1000
Mn-pyCOOH-H_2O	>1000
[Mn-pyCOOH-H_2O]$_n$	>1000

Table S7. Crystal data and structure refinements for the Mn-dipyCO-NO_3.

	**Mn-dipyCO-NO_3**
Empirical formula	$C_{22}H_{15.49}N_4O_8Mn$
Formula weight	546.83
Temperature (K)	100(2)
Wavelength (Å)	0.71073
Crystal system, space group	triclinic, $P\bar{1}$
Unit cell dimensions	
a (Å)	8.1090(3)
b (Å)	9.1350(3)
c (Å)	16.0580(6)
α (°)	102.921(2)
β (°)	103.768(2)
γ (°)	94.868(2)
Cell volume ($Å^3$)	1114.03(7)
Z, Calculated density (g cm^{-3})	2, 1.630
Absorption coefficient (mm^{-1})	0.657
F(000)	567
Crystal size (mm)	0.20 × 0.20 × 0.20
Theta range for data collection (°)	3.08-29.10
Limiting indices	$-11 \le h \le 11$, $-12 \le k \le 12$, $-21 \le l \le 21$
Reflections collected/unique/observed [$I > 2sigma(I)$]	9877/5914 [R_{int} = 0.0199]
Completeness to 2θ (%)	2θ = 25.242°, 99.7
Refinement method	Full-matrix least-squares on F^2
Data/restraints/parameters	5914/0/389
Goodness-of-fit on F^2	1.055
Final *R* indices [I >2sigma(I)]	R_1 = 0.0364, wR_2 = 0.0819
R indices (all data)	R_1 = 0.0482, wR_2 = 0.0889
Largest differences in peak and hole (e $Å^3$)	0.408 and -0.501

4. Supporting references

1. Jabłońska-Wawrzycka, A.; Barszcz, B.; Zienkiewicz, M.; Hodorowicz, M.; Jezierska, J.; Stadnicka, K.; Lechowicz, Ł.; Kaca, W. Eight- and six-coordinated Mn(II) complexes of heteroaromatic alcohol and aldehyde: crystal structure, spectral, magnetic, thermal and antibacterial activity studies. *Spectrochim. Acta Part A* **2014**, *129*, 632–642, doi:10.1016/j.saa.2014.03.105.
2. Jabłońska-Wawrzycka, A.; Zienkiewicz, M.; Barszcz, B.; Rogala, P. Thermoanalytical study of selected transition bivalent metal complexes with 5-carbaldehyde-4-methylimidazole. *J. Therm. Anal. Calorim.* **2012**, *109*, 735–743, doi:10.1007/s10973-012-2329-z.
3. Zienkiewicz, M.; Szlachetko, J.; Lothschütz, C.; Hodorowicz, M.; Jabłońska-Wawrzycka, A.; Sá, J.; Barszcz, B. A novel single-site manganese(II) complex of a pyridine derivative as a catalase mimetic for disproportionation of H_2O_2 in water. *Dalton Trans.* **2013**, *42*, 7761–7767, doi:10.1039/c3dt50288k.
4. Zienkiewicz, M.; Jabłońska-Wawrzycka, A.; Szlachetko, J.; Kayser, Y.; Stadnicka, K.; Sawka-Dobrowolska, W.; Jezierska, J.; Barszcz, B.; Sá, J. Effective catalytic disproportionation of aqueous H_2O_2 with di- and mono-nuclear manganese(II) complexes containing pyridine alcohol ligands. *Dalton Trans.* **2014**, *43*, 8599–8608, doi:10.1039/C3DT53288G.
5. Jabłońska-Wawrzycka, A.; Zienkiewicz, M.; Hodorowicz, M.; Rogala, P.; Barszcz, B. Thermal behavior of manganese(II) complexes with pyridine-2,3-dicarboxylic acid. Spectroscopic, X-ray, and magnetic studies. *J. Therm. Anal. Calorim.* **2012**, *110*, 1367–1376, doi:10.1007/s10973-011-1971-1.
6. Figgis, B.N.; Nyholm, R.S. A convenient solid for calibration of the Gouy magnetic susceptibility apparatus. *J. Chem. Soc.* **1958**, *4190*, 1.
7. König, E.; König, G. *Magnetic Properties of Coordination and Organometallic Transition Metal Compounds*; Hellwege, K.-H., Hellwege, A.M., Eds.; Landolt-Börnstein - Group II Molecules and Radicals; Springer-Verlag: Berlin/Heidelberg, 1966; Vol. 2;.
8. Marken, F.; Neudeck, A.; Bond, A.M.; Stojek, Z. Cyclic Voltammetry; Pulse Voltammetry. In *Electroanalytical methods. Guide to experiments and applications*; Scholz, F., Ed.; Springer Berlin Heidelberg: Berlin, Heidelberg, 2010; pp. 57–119.
9. Otwinowski, Z.; Minor, W. Processing of X-ray diffraction data collected in oscillation mode. *Methods Enzymol.* **1997**, *276*, 307–326, doi:10.1016/S0076-6879(97)76066-X.
10. Nonuis, B. V. Nonius COLLECT, Delft, The Netherlands 1997–2000.
11. Sheldrick, G.M. SHELXL-97, Program for Crystal Structure Refinement, University of Göttingen, Germany 1997.
12. Sheldrick, G.M. SHELXL-17, Program for crystal structure refinement, University of Göttingen, Germany 2017.
13. Sheldrick, G.M. A short history of SHELX. *Acta Crystallogr. Sect. A Found. Crystallogr.* **2008**, *A64*, 112–122.
14. Brandenburg, K.; Putz, H. Diamond - Crystal and Molecular Structure Visualization Crystal Impact. Rathausgasse 30, D-53111 Bonn, GbR, version 3.1 2000.
15. Powder Diffraction File, JCPDS: ICDD, 1601 Park Lane, Swarthmore, PA 19081, Data 1990, File No 40-1290.
16. Wolff, S.K.; Grimwood, D.J.; McKinnon, J.J.; Turner, M.J.; Jayatilaka, D.; Spackman, M.A. Crystal Explorer 2013.
17. Spackman, M.A.; Jayatilaka, D. Hirshfeld surface analysis. *CrystEngComm* **2009**, *11*, 19–32, doi:10.1039/B818330A.
